# SUVR2 is involved in transcriptional gene silencing by associating with SNF2-related chromatin-remodeling proteins in *Arabidopsis*

**DOI:** 10.1038/cr.2014.156

**Published:** 2014-11-25

**Authors:** Yong-Feng Han, Kun Dou, Ze-Yang Ma, Su-Wei Zhang, Huan-Wei Huang, Lin Li, Tao Cai, She Chen, Jian-Kang Zhu, Xin-Jian He

**Affiliations:** 1 National Institute of Biological Sciences, Beijing 102206, China; 2 Department of Horticulture and Landscape Architecture, Purdue University, West Lafayette, IN 47907, USA; 3 Shanghai Center for Plant Stress Biology and Institute of Plant Physiology and Ecology, Shanghai Institutes for Biological Sciences, Chinese Academy of Sciences, Shanghai 200032, China

**Keywords:** SUVR1, SUVR2, CHR19, DNA methylation, chromatin remodeling, transcriptional gene silencing

## Abstract

The SU(VAR)3-9-like histone methyltransferases usually catalyze repressive histone H3K9 methylation and are involved in transcriptional gene silencing in eukaryotic organisms. We identified a putative SU(VAR)3-9-like histone methyltransferase SUVR2 by a forward genetic screen and demonstrated that it is involved in transcriptional gene silencing at genomic loci targeted by RNA-directed DNA methylation (RdDM). We found that SUVR2 has no histone methyltransferase activity and the conserved catalytic sites of SUVR2 are dispensable for the function of SUVR2 in transcriptional silencing. SUVR2 forms a complex with its close homolog SUVR1 and associate with three previously uncharacterized SNF2-related chromatin-remodeling proteins CHR19, CHR27, and CHR28. SUVR2 was previously thought to be a component in the RdDM pathway. We demonstrated that SUVR2 contributes to transcriptional gene silencing not only at a subset of RdDM target loci but also at many RdDM-independent target loci. Our study suggests that the involvement of SUVR2 in transcriptional gene silencing is related to nucleosome positioning mediated by its associated chromatin-remodeling proteins.

## Introduction

DNA methylation, histone H3K9 methylation and other repressive histone marks are involved in transcriptional silencing of transposable elements (TEs) and other DNA repeats^1,2,3^. In *Arabidopsis*, RNA-directed DNA methylation (RdDM) is responsible for *de novo* DNA methylation^4,5^. Two atypical DNA-dependent RNA polymerases IV and V (Pol IV and Pol V) are responsible for producing 24-nt small interfering RNAs (siRNAs) and long noncoding RNAs, respectively, in the RdDM pathway^6^. Pol IV produces single-stranded RNAs, which are converted into double-stranded RNAs by RNA-directed RNA polymerase 2 (RDR2)^2,3,6^. The double-stranded RNAs are cleaved by Dicer Like 3 (DCL3) into 24-nt siRNAs, which are loaded onto AGO4 (Argonaute 4) in the cytoplasm and subsequently transported into the nucleus for the assembly of RdDM effector complex^7,8,9,10^. Pol V generates long noncoding scaffold RNAs to recruit the RdDM effector complex^11,12^. Pol IV and Pol V are multi-subunit RNA polymerases with NRPD1 and NRPE1 being the largest subunits of Pol IV and Pol V, respectively^13,14^. DMS3, DRD1, and RDM1 form the DDR complex and are required for the occupancy of Pol V on chromatin^15,16^. In the end, DRM2 is recruited to RdDM target loci by associating with the AGO4-siRNA complex and mediates *de novo* DNA methylation^17^.

In *Arabidopsis*, there are many SET domain proteins, 15 of which are related to SU(VAR)3-9, including 10 SU(VAR)3-9 homologs SUVH1-SUVH10 and 5 SU(VAR)3-9 related proteins SUVR1-SUVR5^18,19^. The SU(VAR)3-9 homologs SUVH4/KYP, SUVH5, and SUVH6 catalyze histone H3K9 methylation and are responsible for chromatin silencing^5,19,20,21,22,23^. The SUVHs contain an N-terminal YDG/SRA domain in addition to the C-terminal SET domain^18,24^. The SRA domain directly binds methylated DNA and is required for the function of SUVHs in H3K9 methylation^24,25^. SUVH2 and SUVH9 are inactive histone methyltransferases and are responsible for the recruitment of Pol V to chromatin through associating with the DDR complex^26,27^. Different from SUVH1-SUVH10, SUVR1-SUVR5 have no SRA domain^18^. SUVR4 is an active histone methyltransferase with a preference for H3K9me1^28^. The N-terminal WIYLD domain of SUVR4 binds ubiquitin and facilitates the conversion of H3K9me1 to H3K9me3^29^. SUVR5 is involved in H3K9 methylation *in vivo*, although no histone methytransferase activity was detected for SUVR5 by *in vitro* assays^30^.

Several chromatin-remodeling proteins were previously demonstrated to be involved in DNA methylation in *Arabidopsis*^15,31,32,33,34^. DDM1 is an SWI2/SNF2 chromatin-remodeling protein required for maintaining DNA methylation at the whole-genome level^31,34^. The SNF2 chromatin-remodeling protein DRD1 can associate with DMS3 and RDM1 and forms a complex required for the recruitment of Pol V to chromatin^15,16^. The SNF2 chromatin-remodeling protein CLSY1 associates with Pol IV and is required for the accumulation of Pol IV-dependent siRNAs^33,35,36,37^.

Chromatin-remodeling proteins can also affect epigenetic silencing independently of DNA methylation. MOM1 (morpheus molecule 1), a CHD3-like chromatin-remodeling protein, is a transcriptional silencing regulator that does not affect DNA methylation^38^. MOM1 acts in transcriptional silencing independently of the RdDM pathway, however, it enhances transcriptional silencing at certain RdDM target loci^39,40^. MORC1 (Microrchidia 1) and MORC6/DMS11 are two members of the conserved MORC adenosine triphosphatase (ATPase) family^41,42^. MORC1 and MORC6 are not only involved in RdDM but also are required for heterochromatin condensation^26,41,42,43^. The function of MORC1 and MORC6 in heterochromatin condensation is at least partially responsible for transcriptional gene silencing in *Arabidopsis*. IDN2 is a canonical RdDM component that associates with its paralogs and forms a complex required for RdDM^44,45,46^. IDN2 was thought to be recruited to RdDM target loci by Pol V-produced long noncoding RNAs and tether the SWI/SNF chromatin-remodeling complex to the loci, thereby mediating nucleosome positioning and transcriptional gene silencing^47^.

In this study, we identified SUVR2 as a regulator of transcriptional gene silencing by a forward genetic screen. The silencing of the *RD29A-LUC* transgene in the DNA demethylase mutant *ros1* and the silencing of endogenous transposable elements is suppressed by *suvr2*, while the DNA methylation of these loci is either unchanged or slightly decreased. Recently, SUVR2 was reported as a component of the RdDM pathway^5^. Our study demonstrates that SUVR2 forms a complex with its homolog SUVR1 and associates with the SNF2-related chromatin-remodeling proteins CHR19, CHR27, and CHR28, thereby mediating nucleosome positioning and transcriptional silencing. The study reveals a novel mechanism underlying transcriptional gene silencing, which may be conserved in eukaryotic organisms.

## Results

### Identification of SUVR2 as a regulator of transcriptional gene silencing

**Figure 1:**
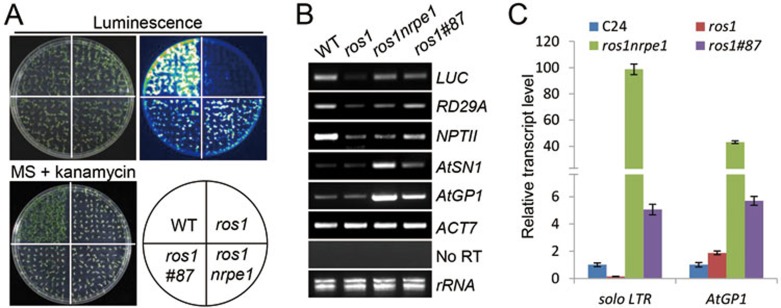
SUVR2 is required for transgene and transposable element silencing. **(A)** The luminescence phenotype and kanamycin resistance of WT, *ros1*, *ros1#87*, and *ros1nrpe1*. The *Arabidopsis* seedlings were cold-treated at 4 °C for 2-4 days and subjected to luminescence imaging. Kanamycin sensitivity assay was carried out on MS medium supplemental with 150 mg/l kanamycin. **(B)** Semiquantitative RT-PCR was carried out to detect the RNA transcript levels of *RD29A-LUC*, endogenous *RD29A*, *35S-NPTII*, and transposable elements in WT, *ros1*, *ros1nrpe1*, and *ros1#87*. The actin gene *ACT7* was amplified as an internal control. No RT shows amplification of *ACT7* using RNA samples as templates without reverse transcription, indicating no DNA contamination in the RNA samples. **(C)** The RNA transcript levels of *solo LTR* and *AtGP1* as determined by quantitative RT-PCR.

A robust *RD29A* promoter-driven luciferase transgene (*RD29A-LUC*) system in the *ros1* (*repressor of silencing 1*) mutant background was previously developed and used for screening for cellular factors required for transcriptional gene silencing^48,49,50,51^. *ROS1* encodes a DNA demethylase in *Arabidopsis*, and loss-of-function mutations in *ROS1* lead to hypermethylation of many genomic loci^52,53^. The stress-inducible *RD29A-LUC* transgene is expressed in the wild-type background after cold treatment and the plants emit bright luminescence, whereas the transgene is silenced in *ros1* mutant plants due to hypermethylation of the transgenic *RD29A* promoter (Figure 1A and 1B)^48^. In this transgenic line, there is another transgene, the *35S* promoter-driven kanamycin resistance gene *NPTII* (*35S-NPTII*), which is also highly expressed in the wild type and is silenced in *ros1* (Figure 1A and 1B)^48^. The *ros1* mutant plants harboring the *RD29A-LUC* and *35S-NPTII* transgenes were previously mutagenized by T-DNA transformation or EMS treatment and the mutagenized populations were used for screening for suppressors of *ros1*^51,52,53,54^. The mutants with reactivated expression of either *RD29A-LUC* and/or *35S-NPTII* were subjected to map-based cloning. Most of the known RdDM components were recovered and disruption of RdDM suppressed the silencing of *RD29A-LUC* but not *35S-NPTII*^55^.

A *ros1#87* mutant was recovered in this screen, and the luminescence intensity is much higher in *ros1#87* than in *ros1* (Figure 1A), indicating that the silencing of the *RD29A-LUC* transgene is suppressed by the *#87* mutation. This effect of *#87* is slightly weaker than that of the Pol V mutation *nrpe1* (Figure 1A). The kanamycin resistance is slightly higher in *ros1#87* than that in *ros1*, whereas it is comparable between *ros1nrpe1* and *ros1* (Figure 1A). We performed RT-PCR to test the effect of the *#87* mutation on the expression of *RD29A-LUC*. The *RD29A-LUC* transgene and endogenous *RD29A* gene are silenced in *ros1* but reactivated in *ros1#87*, with *ros1nrpe1* serving as a positive control (Figure 1B). The *35S-NPTII* transgene is silenced in *ros1* and is partially reactivated in *ros1#87* but is not reactivated in *ros1nrpe1* (Figure 1B), which is consistent with the kanamycin resistance phenotypes of the mutants as seen in Figure 1A. The transposable elements *AtSN1*, *AtGP1*, and *solo LTR* are typical RdDM target loci^9,56,57^. We found that the transcript levels of these loci are increased in *ros1nrpe1* and to a lesser extent in *ros1#87* (Figure 1B and 1C). These data suggest that the *#87* mutation affects transcriptional gene silencing at RdDM target loci.

We performed map-based cloning using the F2 segregating population from a cross between the *ros1#87* mutant in the C24 background and the *ros1-4* mutant (Salk_045303) in the Col-0 background. The *#87* mutation was mapped to a ∼1 Mb region on chromosome 5 (Supplementary information, Figure S1A). We sequenced the candidate genes in this region and found a G-to-A mutation at the splice receptor site of the *AT5G43990* (*SUVR2*) second intron (Supplementary information, Figure S1B). The mutation causes a frame shift in the *SUVR2* coding sequence in the *ros1#87* mutant (Supplementary information, Figure S1C). SUVR2 is a member of the *Drosophila* SU(VAR)3-9 related proteins and contains three conserved domains, which are the N-terminal WIYLD domain, the C-terminal Pre-SET and SET domains (Supplementary information, Figure S1B). To determine whether the mutation in *SUVR2* is responsible for the silencing of the *RD29A-LUC* transgene, we transformed a construct harboring the full-length *SUVR2* genomic sequence into the *ros1#87* mutant and found that the silencing defect of *RD29A-LUC* in *ros1#87* is complemented by the *SUVR2* construct in several individual *SUVR2* transgenic lines (Supplementary information, Figure S1D). The results suggest that the G-to-A mutation in *SUVR2* is responsible for the defective silencing phenotype of *ros1#87*. Thus, *#87* is thereafter referred to as *suvr2*.

### SUVR2 has both RdDM-dependent and -independent roles in transcriptional gene silencing

**Figure 2:**
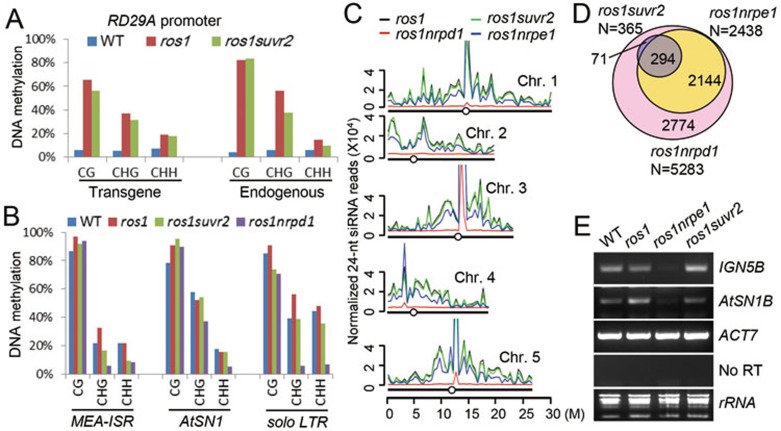
The function of SUVR2 in transcriptional silencing is partially independent of RdDM. **(A**, **B)** DNA methylation was determined by bisulfite sequencing at the promoters of transgenic *RD29A-LUC* and endogenous *RD29A*, *MEA-ISR*, *AtSN1*, and *solo LTR*. The percentages of CG, CHG, and CHH methylation are separately shown. **(C)** Plots indicate the distribution of Pol IV-dependent 24-nt siRNA across the five *Arabidopsis* chromosomes in *ros1*, *ros1suvr2*, *ros1nrpd1*, and *ros1nrpe1*. **(D)** Venn diagram shows the numbers of affected 24-nt siRNA regions and their overlaps in *ros1suvr2*, *ros1nrpd1*, and *ros1nrpe1* relative to *ros1*. **(E)** Accumulation of Pol V-produced noncoding RNAs from *AtSN1B* and *IGN5B* was detected by semiquantitative RT-PCR. The dependence of the noncoding RNAs on Pol V was shown in *ros1nrpe1* as a control.

We performed locus-specific bisulfite sequencing to determine whether the effect of *suvr2* on transcriptional gene silencing is due to a reduction in DNA methylation. Our data demonstrated that the DNA methylation levels of the transgenic and endogenous *RD29A* promoters are low in wild-type plants, whereas they are obviously increased in *ros1* (Figure 2A). The DNA methylation levels of the transgenic and endogenous *RD29A* promoters are either not affected or slightly decreased in *ros1suvr2* compared to those in *ros1* (Figure 2A). As reported previously^54^, DNA methylation at the promoter of *RD29A-LUC* is markedly reduced by the RdDM mutation *nrpe1*. Although *suvr2* has no significant effect on DNA methylation at the transgenic *RD29A* promoter, the silencing of *RD29A-LUC* is markedly released by *suvr2* (Figure 1A and 1B). These results suggest that the action of SUVR2 in the silencing of *RD29A-LUC* and endogenous *RD29A* is at least partially independent of DNA methylation. *AtSN1*, *solo LTR* and *MEA-ISR* are RdDM target loci^9,57,58^. Our result indicated that DNA methylation of the three loci is markedly decreased by the Pol IV mutation *nrpd1* in the *ros1nrpd1* mutant (Figure 2B). In the *ros1suvr2* mutant, DNA methylation is slightly decreased at *MEA-ISR* and *solo LTR* but not at *AtSN1* (Figure 2B) even though the transcript level of *AtSN1* is clearly increased (Figure 1B). Thus, the involvement of SUVR2 in the silencing of *AtSN1* is independent of DNA methylation. These data suggest that *suvr2* affects DNA methylation at a subset of RdDM target loci, but the defective transcriptional silencing caused by *suvr2* is not necessarily coupled with a decrease in DNA methylation. Thus, the involvement of SUVR2 in transcriptional gene silencing is likely partially through a DNA methylation-independent mechanism.

Although the *suvr2* mutation affects DNA methylation at the RdDM target loci *MEA-ISR* and *solo LTR*, the effect of *suvr2* is much weaker than that of the canonical RdDM mutation *nrpd1* (Figure 2B). To confirm the effect of *suvr2* on DNA methylation, we tested DNA methylation at another RdDM target locus, *IGN23*. For analysis of the *IGN23* DNA methylation, genomic DNA was digested by the DNA methylation-sensitive restriction enzyme HaeIII followed by quantitative PCR. The result indicated that the DNA methylation level of *IGN23* is significantly reduced in *ros1suvr2* relative to *ros1* (Supplementary information, Figure S2). A full-length *SUVR2* construct was transformed into *ros1suvr2* to determine whether the *SUVR2* transgene rescues the defect in DNA methylation. In two individual *SUVR2* transgenic lines, the *IGN23* methylation level is not only higher than that in *ros1suvr2* but also in the wild type and *ros1* (Supplementary information, Figure S2). These results demonstrates that SUVR2 is necessary for DNA methylation at a subset of RdDM target loci.

Small RNA deep sequencing was performed to determine whether *suvr2* affects transcriptional silencing by attenuating Pol IV-produced 24-nt siRNAs. The deep sequencing data indicated that the Pol IV mutation *nrpd1* results in a severe reduction of 24-nt siRNAs throughout all 5 chromosomes while the Pol V mutation *nrpe1* leads to an obvious reduction of 24-nt siRNAs at two arms of each chromosome but has little effect on centromeric regions (Figure 2C). However, the overall abundance of Pol IV-dependent 24-nt siRNAs in *ros1suvr2* is similar to that in *ros1* (Figure 2C, Supplementary information, Table S1). We counted the numbers of 24-nt siRNA regions, in which the 24-nt siRNAs are downregulated in *ros1suvr2*, *ros1nrpd1*, and *ros1nrpe1* compared to *ros1* (Figure 2C, Supplementary information, Table S1). 24-nt siRNAs from 5 283 siRNA regions are significantly downregulated in *ros1nrpd1* compared to *ros1*, among which siRNAs from 2 438 regions are also downregulated in *ros1nrpe1* (Figure 2D, Supplementary information, Table S1). Different from the RdDM mutants *nrpd1* and *nrpe1*, *suvr2* only affects 365 24-nt siRNA regions, which are 7% of the NRPD1 targets. 294 of the 365 regions overlap with the NRPE1 targets, while 71 overlap with NRPD1 targets but are independent of NRPE1 (Figure 2D). Our data demonstrate that the effect of *suvr2* on 24-nt siRNA accumulation is much more limited than that of *nrpe1*, which is different from a previous study reporting that *suvr2* and *nrpe1* have a similar effect on 24-nt siRNA accumulation^5^.

Pol V produces long noncoding RNAs, which are responsible for the assembly of RdDM effector complex^11^. We tested whether *suvr2* affects Pol V-produced noncoding RNAs at the *IGN5B* and *AtSN1B* sites. The *IGN5B* and *AtSN1B* transcripts are blocked by the Pol V mutation *nrpe1* but are not affected by *suvr2* (Figure 2E), indicating that SUVR2 is not required for Pol V transcription. The result suggests that the action of SUVR2 is either at a step downstream of Pol V transcription in the RdDM pathway or independent of RdDM.

To investigate the relationship between SUVR2 and the RdDM pathway, we determined whether SUVR2 interacts with major RdDM components. Because SUVR2 is not required for the accumulation of most Pol IV-dependent siRNAs (Figure 2C and 2D, Supplementary information, Table S1), it is unlikely involved in Pol IV-dependent siRNA biogenesis at the early steps of the RdDM pathway. Thus, only the downstream RdDM components NRPE1, AGO4, DMS3, RDM1, KTF1, and DRM2 were selected for the interaction assay. By co-IP, we found that none of these RdDM components interact with SUVR2 (Supplementary information, Figure S3A-S3F). We further performed nuclear coimmunolocalization assays to determine whether SUVR2 colocalizes with DRM2, NRPE1, or KTF1 in the nucleus. The results indicated that SUVR2 forms a few condensed foci in the nucleus but does not significantly overlap with the signals of DRM2, NRPE1, and KTF1 (Supplementary information, Figure S4A-S4C).

**Figure 3:**
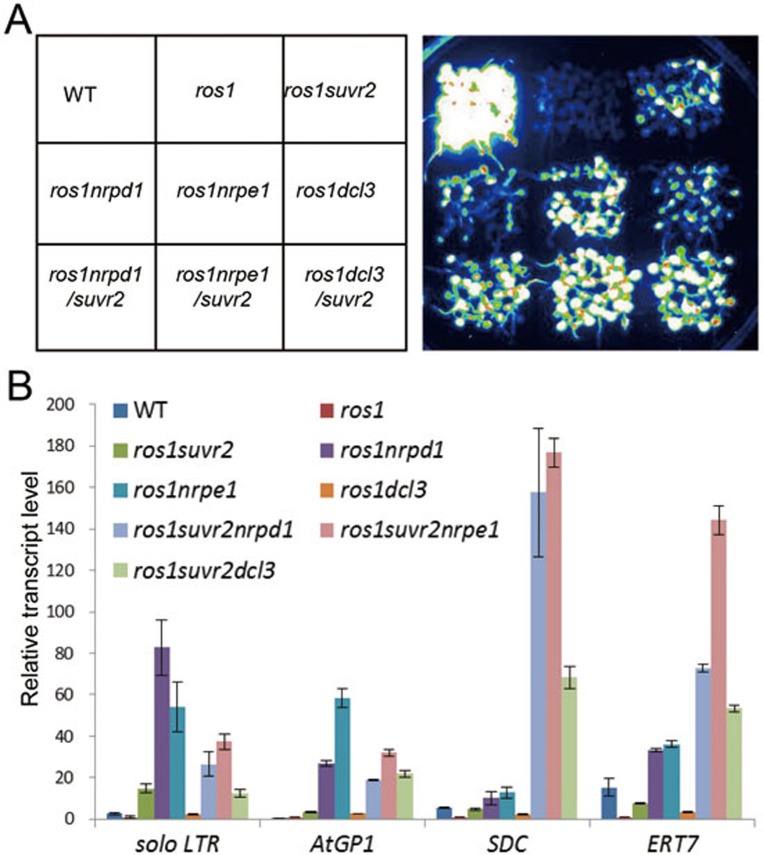
The genetic relationship between SUVR2 and RdDM components. **(A)** The expression of *RD29A-LUC* transgene is indicated by luminescence imaging. The luminescence images are shown for the wild type, *ros1*, *ros1suvr2*, *ros1nrpd1*, *ros1nrpe1*, and *ros1dcl3*, as well as for the indicated mutants in which the *suvr2* mutation was combined with each of the RdDM mutations. **(B)** The transcript levels of the endogenous RdDM target loci *solo LTR*, *AtGP1*, *SDC*, and *ERT7* were determined by quantitative RT-PCR. The experiments were biologically repeated for three times.

Many RdDM components were identified from the *ros1* suppressor screen, including the core components NRPD1, NRPE1 and DCL3 (Figure 3A). To examine the genetic relationship between SUVR2 and the RdDM pathway, *ros1suvr2* was crossed to *ros1nrpd1*, *ros1nrpe1*, and *ros1dcl3* to combine *suvr2* with RdDM mutations. Luminescence images show that the expression of the *RD29A-LUC* transgene is suppressed in *ros1* but reactivated by *suvr2*, *nrpd1*, *nrpe1* and *dcl3* (Figure 3A). The *RD29A-LUC* expression level in *ros1suvr2* is comparable to that in *ros1nrpd1* and *ros1dcl3* but weaker than that in *ros1nrpe1* (Figure 3A). In *ros1nrpd1suvr2* and *ros1dcl3suvr2*, the *RD29A-LUC* expression is synergistically enhanced as compared with that in *ros1nrpd1*, *ros1dcl3*, and *ros1suvr2* (Figure 3A). In contrast, the *RD29A-LUC* expression is less enhanced in *ros1nrpe1suvr2* relative to that in *ros1nrpe1* (Figure 3A). We performed quantitative RT-PCR to determine the transcript levels of the endogenous RdDM target loci *solo LTR*, *AtGP1*, *SDC*, and *ERT7* (Figure 3B). The results indicated that the silencing of all these loci is relieved by *suvr2*, *nrpd1*, *nrpe1*, and *dcl3* (Figure 3B). At these RdDM target loci, the effect of *suvr2* and *dcl3* is comparable but is weaker than that of *nrpd1* and *nrpe1*. The weak effect of *dcl3* is consistent with the functional redundancy between DCL3 and its close homologs DCL2 and DCL4 in RdDM^59^. The transcript levels of *solo LTR* and *AtGP1* are not increased by *suvr2* in *ros1suvr2nrpd1*, *ros1suvr2nrpe1*, but are increased by *suvr2* in *ros1suvr2dcl3* (Figure 3B). Meanwhile, at *SDC* and *ERT7* sites, the effect of *suvr2* is synergistic with that of all the RdDM mutations (Figure 3B), suggesting that the involvement of SUVR2 in the silencing of *SDC* and *ERT7* is at least partially through an RdDM-independent pathway. These results suggest that the interplay between SUVR2 and the RdDM pathway is in a locus-specific manner.

**Figure 4:**
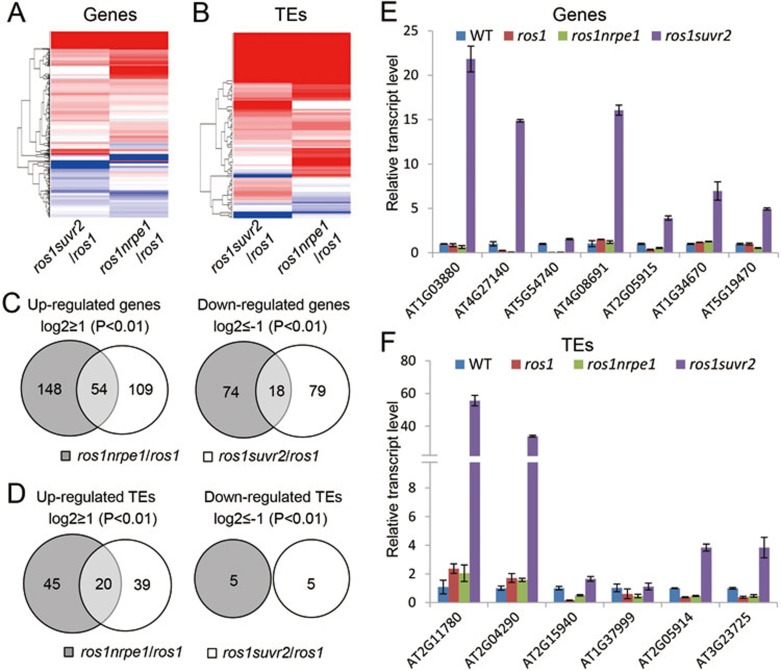
The effect of *suvr2* and *nrpe1* on RNA transcript levels as determined by RNA-seq. **(A**, **B)** Heat maps indicate differentially expressed genes **(A)** and TEs **(B)** in *ros1suvr2* and *ros1nrpe1* relative to *ros1*. Red and blue bars represent upregulated and downregulated genes or TEs, respectively. **(C**, **D)** Differentially expressed genes **(C)** or TEs **(D)** were compared between *ros1suvr2* and *ros1nrpe1*. Differentially expressed genes or TEs were defined when log_2_(fold changes of normalized reads) ≥ 1 or ≤ −1 and *P* value < 0.01. **(E**, **F)** Quatitative RT-PCR was performed to confirm SUVR2-specific target genes **(E)** and TEs **(F)** identified by RNA-seq.

We performed RNA-seq to compare the effect of *suvr2* and *nrpe1* on the transcript levels of genes and TEs at the whole-genome level. 5.9 × 10^7^, 5.6 × 10^7^, and 5.0 × 10^7^ reads were obtained from the libraries of *ros1*, *ros1nrpe1*, and *ros1suvr2*, respectively (Supplementary information, Table S2). More than 97% of the reads were mapped to the *Arabidopsis* genome sequence. From the RNA-seq data, we found that many genes and TEs are coregulated by *suvr2* and *nrpe1* (Figure 4A-4D, Supplementary information, Tables S3,S4,S5,S6). The numbers of upregulated TEs in *suvr2* and *nrpe1* are much higher than those of downregulated ones (Figure 4B and 4D), which is consistent the function of SUVR2 and NRPE1 in the silencing of TEs. The RNA-seq data indicated that 163 genes and 59 TEs are significantly upregulated by *suvr2* (Figure 4C and 4D, Supplementary information, Tables S3 and S5). Among them, 32.9% (54/163) of genes and 33.9% (20/59) of TEs overlap with the upregulated genes and TEs caused by *nrpe1*, respectively (Figure 4C and 4D, Supplementary information, Tables S3,S4,S5,S6). The overlapping of upregulated genes and TEs in *suvr2* and *nrpe1* is significantly higher than expected by chance (*P* < 0.01), suggesting that SUVR2 can act at a subset of RdDM target loci. Additionally, about two-third of upregulated genes and TEs caused by *suvr2* do not overlap with those caused by *nrpe1* (Figure 4A-4D, Supplementary information, Tables S3,S4,S5,S6). These results support the inference that SUVR2 has both RdDM-dependent and -independent roles in transcriptional gene silencing.

We randomly selected the genes and TEs that are specifically upregulated by *suvr2* as determined by our RNA-seq experiment (Figure 4A-4D, Supplementary information, Tables S3,S4,S5,S6) to confirm their expression by quantitative RT-PCR (Figure 4E and 4F). The results suggest that most of the selected loci (7/8 for genes and 6/7 for TEs) are significantly up-regulated by *suvr2* but either not affected or weakly affected by *nrpe1* (Figure 4E and 4F). Because the *nrpe1* mutation was thought to completely block the RdDM pathway, the finding of the SUVR2-specific target loci demonstrates that SUVR2 has an RdDM-independent role in transcriptional gene silencing in addition to its role in the RdDM pathway.

### The function of SUVR2 in transcriptional gene silencing is independent of histone H3K9 methylation

**Figure 5:**
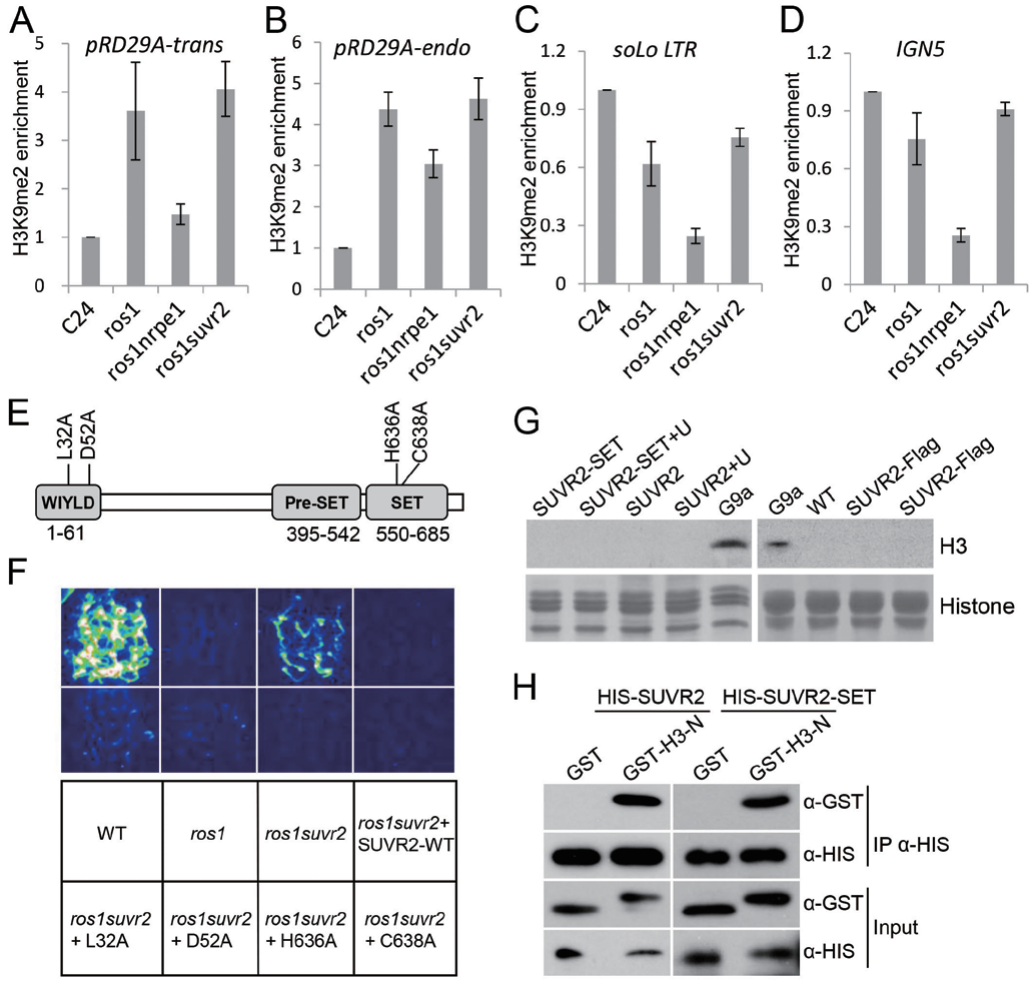
SUVR2 is involved in transcriptional gene silencing independently of the histone H3K9 methyltransferase activity. **(A**-**D)** The H3K9me2 levels at the promoters of *RD29A-LUC* **(A)** and endogenous *RD29A* **(B)**, *solo LTR* **(C)**, and *IGN5* **(D)** were determined by ChIP-PCR in the wild type, *ros1*, *ros1suvr2*, and *ros1nrpe1*. The protein-coding gene *ACT7* that has a low H3K9me2 level was used as an internal control. **(E)** The diagram of the SUVR2 protein indicates that SUVR2 contains three conserved domains: WIYLD, Pre-SET, and SET. The conserved residues that have been subjected to point mutation in this study are shown. **(F)** The complementation test by using wild-type and mutated SUVR2 transgenes. The constructs harboring wild-type and mutated *SUVR2* sequences were introduced into *ros1suvr2*. The luminescence image indicates the expression of *RD29A-LUC*. In the mutated *SUVR2* sequences, L32 and D52 in the WIYLD domain, and H636 and C638 in the SET domain are mutated to alanine. **(G)** The SUVR2 protein was purified from bacteria and *Arabidopsis* and was used in the *in vitro* histone methyltransferase activity assay. The human SU(VAR)3-9 homolog G9a that shows active histone H3K9 methylation was used as a positive control. “U” is ubiquitin. **(H)** The interaction of SUVR2 with the histone H3 peptide as determined by pull-down assay. The full-length SUVR2 and the SUVR2 fragment containing the PreSET and SET domains were bacterially expressed in fusion with the His tag. The N-terminus of the histone H3 was expressed in fusion with the GST tag in bacteria.

The above bisulfite sequencing result suggests that DNA methylation at the promoters of *RD29A-LUC* and endogenous *RD29A* is not significantly affected by *suvr2* (Figure 2A). We performed a ChIP assay to determine the effect of *suvr2* on the repressive histone mark H3K9me2. The result indicated that the H3K9me2 levels at the transgenic and endogenous *RD29A* promoters are increased by *ros1* (Figure 5A and 5B). This effect of *ros1* is consistent with previous reports^60,61^. In the *ros1* mutant background, the enrichment of H3K9me2 on the transgenic and endogenous *RD29A* promoters is clearly reduced by *nrpe1* (Figure 5A and 5B), which is consistent with the previous reports showing that *nrpe1* affects H3K9me2 at RdDM target loci^11,57^. However, *suvr2* has no effect on H3K9me2 at the transgenic and endogenous *RD29A* promoters (Figure 5A and 5B). Moreover, we found that the enrichment of H3K9me2 at the endogenous RdDM target loci *solo LTR* and *IGN5* is reduced by *nrpe1* but not by *suvr2* (Figure 5C and 5D). These results suggest that the function of SUVR2 in transcriptional gene silencing is independent of H3K9me2. Moreover, we performed H3K9me3 ChIP to determine the H3K9me3 levels at the RdDM target loci *solo LTR*, *IGN5*, and *IGN23*. The results indicated that the enrichment of H3K9me3 relative to input at the RdDM target loci (∼0.03%-0.05% at *solo LTR*, ∼0.015%-0.025% at *IGN5*, and ∼0.01% at *IGN23*) is much lower than that at the protein-coding gene *ACT2* (∼0.09%) (Supplementary information, Figure S5), which is consistent with the previous studies reporting that H3K9me3 is preferentially associated with euchromatic protein-coding genes but not with heterochromatic transposable elements^62,63^. The H3K9me3 levels at *solo LTR*, *IGN5*, and *IGN23* are not reduced by *nrpe1* and *suvr2* (Supplementary information, Figure S5). Thus, H3K9me3 is unlikely to play a role in transcriptional gene silencing at transposable elements targeted by SUVR2.

SUVR2 is a SU(VAR)3-9-related protein and contains three annotated domains, which are the WIYLD domain, the Pre-SET domain, and the SET domain (Figure 5E). The WIYLD domain is conserved in the SUVR family in *Arabidopsis*^28^. The WIYLD domain of SUVR4 binds ubiquitin and affects the histone methyltransferase activity of SUVR4^29^. The SUVR4 residue D74 in the WIYLD domain is required for the binding of SUVR4 to ubiquitin^29^. The SUVR2 residue D52 corresponds to the SUVR4 residue D74 (Figure 5E, Supplementary information, Figure S6A). The SET domain is conserved in SU(VAR)3-9 homologs (Supplementary information, Figure S6B). Previous structural and biochemical studies indicated that critical residues in the SET domain of SU(VAR)3-9 homologs are essential for histone H3K9 methyltransferase activity^20,64^. The SUVR2 residues H636 and C638 correspond to the essential catalytic residues in the SU(VAR)3-9 family proteins (Figure 5E, Supplementary information, Figure S6B).

To determine whether the critical residues in the WIYLD domain and the SET domain are required for the function of SUVR2 in transcriptional gene silencing, we generated SUVR2 constructs harboring the WIYLD domain mutations L32A and D52A, and the SET domain mutations H636A and C638A, and transformed the constructs into *ros1suvr2* to investigate whether the mutated SUVR2 can rescue the defective transcriptional gene silencing of *ros1suvr2*. Luminescence imaging indicated that the silencing of *RD29A-LUC* was restored by the four mutated SUVR2 sequences, as well as by the wild-type SUVR2 sequence in *ros1suvr2* (Figure 5F). Quantitative RT-PCR indicated that the silencing of both *RD29A-LUC* and endogenous *RD29A* is not affected by the mutations in SUVR2 (Supplementary information, Figure S7). Moreover, we checked the transcript level of the endogenous transposable element *AtGP1* and found that the silencing of *AtGP1* is restored by both wild-type and mutated *SUVR2* transgenes (Supplementary information, Figure S7). These results suggest that the SUVR2 mutations do not affect the function of SUVR2 in transcriptional gene silencing. Thus, none of the four sites that were mutated in the WIYLD and SET domains is required for the function of SUVR2 in transcriptional gene silencing, suggesting that the involvement of SUVR2 in transcriptional gene silencing is independent of ubiquitin binding and histone methyltransferase activity.

Histone methyltransferase activity was not detected for SUVR1 and SUVR2 in a previous study^28^. We tested the histone methyltransferase activity of SUVR2 using the SUVR2 proteins purified from both bacteria and *Arabidopsis*. Neither the full-length SUVR2 nor the SET domain of SUVR2 can methylate core histones from calf thymus, whereas the human SU(VAR)3-9 homolog, G9a, can effectively methylate the histones (Figure 5G, left panel). Since addition of ubiquitin can affect the histone methyltransferase activity of SUVR4^29^, we examined whether ubiquitin may stimulate the histone methyltransferase activity of SUVR2. The results showed that no activity was detected even when ubiquitin was added (Figure 5G, left panel). The histone methyltransferase activity of SUVR2 was not detected even when SUVR2 was expressed in *Arabidopsis* (Figure 5G, right panel). These results suggest that SUVR2 is not an active histone methyltransferase and is involved in transcriptional gene silencing through an unknown mechanism.

Although SUVR2 fails to methylate histone H3 at lysine 9, it is still possible that SUVR2 is capable of binding the histone H3 peptide. The full-length SUVR2 and the truncated SUVR2 containing the PreSET and SET domains were purified from bacteria and used to test whether they bind the histone H3 peptide. The results indicated that both full-length and truncated SUVR2 proteins bind the N-terminus of H3 as determined by the *in vitro* pull-down assay (Figure 5H), suggesting that the PreSET and SET domains of SUVR2 may associate with chromatin through binding to H3.

### SUVR2 forms a complex with SUVR1 and associates with CHR19, CHR27, and CHR28

**Figure 6:**
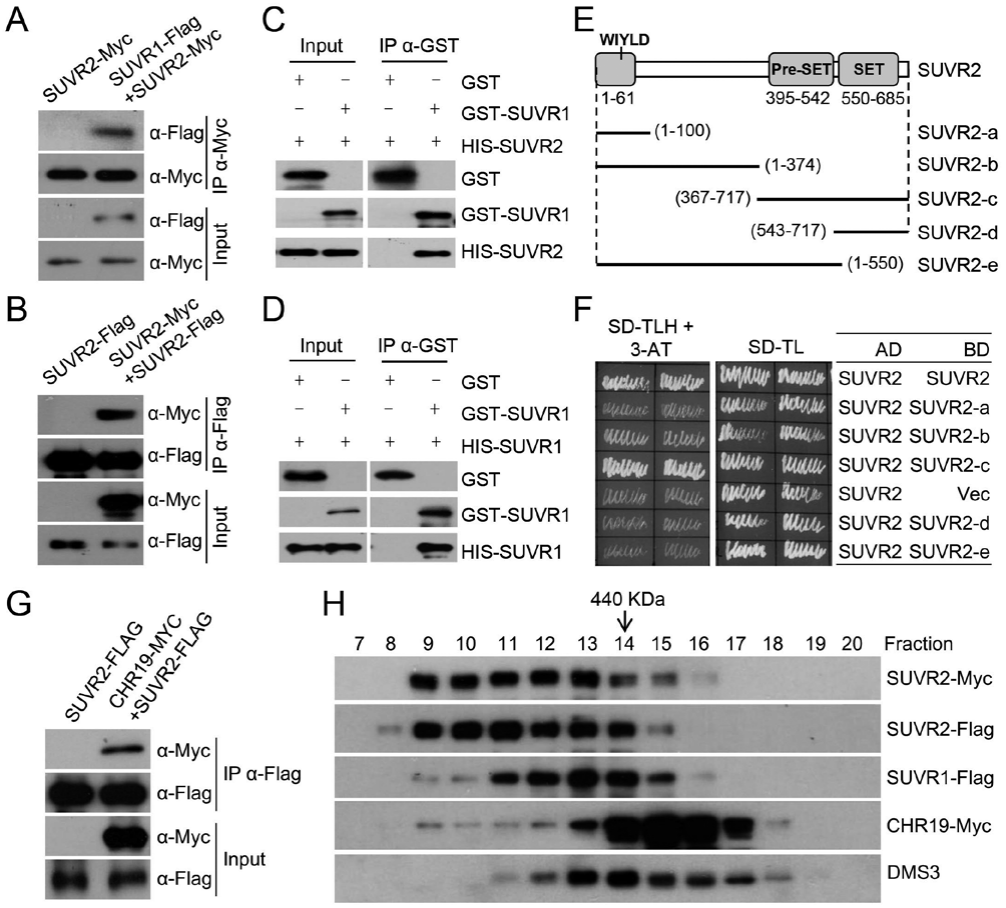
SUVR2 interacts with SUVR1 and the SNF2 chromatin-remodeling protein CHR19. **(A)** The interaction between SUVR1 and SUVR2 as determined by co-IP. The protein extract was isolated from the plants that express both *SUVR1-Flag* and *SUVR2-Myc* transgenes. The extract was immunoprecipitated by anti-Myc antibody and subjected to western blotting. **(B)** The interaction between SUVR2 and SUVR2 was determined by co-IP. The *SUVR2-Myc* transgene was combined with the *SUVR2-Flag* transgene in *Arabidopsis*. **(C**, **D)** GST pull-down assay was performed to test the interaction between GST-SUVR1 and HIS-SUVR2 **(C)** or HIS-SUVR1 **(D)**. The GST protein was expressed and used as a control. **(E)** Diagrams of the full-length and truncated SUVR2 protein sequences in the pGBKT7 vector. The constructs were used in yeast two-hybrid assay. **(F)** The yeast strains harboring the indicated constructs were grown on both SD-TL and SD-TLH (the synthetic dropout medium minus Trp, Leu, and His). 20 mM 3-AT was added in SD-TLH to inhibit the growth of yeast strains. **(G)** The interaction of SUVR2 with CHR19 was determined by co-IP. The *CHR19-Myc* transgene was combined with the *SUVR2-Flag* transgene in *Arabidopsis*. **(H)** The elution profiles of SUVR2-Myc, SUVR2-Flag, SUVR1-Flag, CHR19-Myc, and DMS3 in gel filtration assay. Anti-Myc, anti-Flag antibody, and DMS3-specific antibody were used in western blotting.

To understand how SUVR2 is involved in transcriptional gene silencing, we tried to isolate SUVR2-interacting proteins by affinity purification of SUVR2-Flag in *SUVR2-Flag* transgenic plants. Mass spectrometric data indicated that SUVR2-Flag copurified with its close homolog SUVR1 and three SNF2 ATP-dependent chromatin-remodeling proteins including CHR19, CHR27 and CHR28 (Table 1, Supplementary information, Table S7). To confirm the interaction of SUVR2 with SUVR1, CHR19, and CHR27, we generated transgenic plants harboring *SUVR1-Flag*, *CHR19-Myc*, or *CHR27-Myc* transgene, and used these plants to perform affinity purification of SUVR1-Flag, CHR19-Myc, and CHR27-Myc, respectively. Mass spectrometric analysis indicated that SUVR2 is copurified with SUVR1-Flag, CHR19-Myc, and CHR27-Myc (Table 1, Supplementary information, Table S7). Co-IP experiments demonstrated that SUVR2 interacts with SUVR1 as well as with itself (Figure 6A and 6B). We performed GST pull-down assays to determine whether SUVR1 and SUVR2 may directly interact with each other. The results indicated that bacterially expressed GST-SUVR1 can pull down both His-SUVR2 and His-SUVR1 (Figure 6C and 6D), confirming that SUVR1 is able to form a complex with SUVR1 or SUVR2. Unexpectedly, we found that GST-SUVR2 and His-SUVR2 is unable to pull down each other (Supplementary information, Figure S8A and S8B). However, the interaction between two SUVR2 proteins was confirmed by a yeast two-hybrid assay (Figure 6E and 6F). It is possible that some modifications that are required for the SUVR2-SUVR2 interaction are absent in the bacterially expressed SUVR2.

To identify the domain that is required for the SUVR2-SUVR2 interaction, we produced constructs harboring truncated *SUVR2* versions and separately introduced the constructs into yeast for two-hybrid assays (Figure 6E and 6F). The results indicated that the truncated sequences containing both PreSET and SET domains can interact with SUVR2 and the absence of either domain causes a loss of the interaction (Figure 6E and 6F, Supplementary information, Figure S9), suggesting that the PreSET and SET domains are not only necessary but also sufficient for the interaction between two SUVR2 proteins.

We introduced both *SUVR2-Flag* and *CHR19-Myc* into *Arabidopsis* to determine the interaction between SUVR2 and CHR19 by co-IP. The result demonstrated that SUVR2 interacts with CHR19 (Figure 6G). In gel filtration, SUVR1 and SUVR2 were largely coeluted in the fractions of > 440 KDa (Figure 6H), indicating that the two proteins exist in a tight complex *in vivo*. SUVR2 and CHR19 show overlapping but different elution patterns in gel filtration (Figure 6H), suggesting that the interaction between SUVR2 and CHR19 is probably spatiotemporally specific *in vivo*.

**Figure 7:**
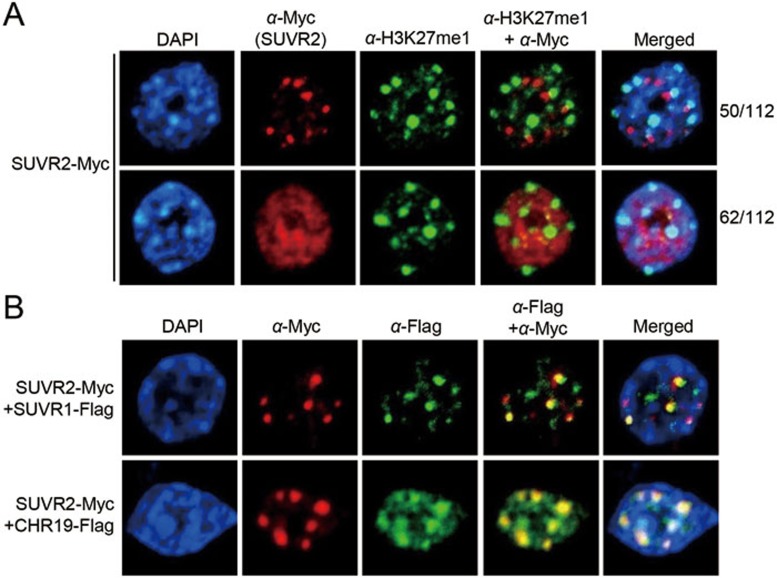
SUVR2 colocalizes with SUVR1 and CHR19 in the nucleus. **(A)** The SUVR2-Myc signals are shown in nuclei. The SUVR2-Myc signals were detected by anti-Myc antibody. Anti-H3K27me antibody was used for immunostaining to indicate heterochromatin foci. The nuclei were stained by DAPI as a control. **(B)** Coimmunolocalization was performed to determine whether SUVR2 is colocalized with SUVR1 and CHR19. *SUVR1-Flag* and *CHR19-Flag* constructs were separately introduced into the *SUVR2-Myc* transgenic plants. The nuclei were extracted from the offspring plants and subjected to coimmunostaining with both anti-Myc antibody and anti-Flag antibody.

Nuclear coimmunolocalization assays were performed to test whether SUVR2 colocalizes with SUVR1 and CHR19. SUVR2 shows two nuclear localization patterns with one forming condensed foci (50/112) and the other showing diffused signals (62/112) (Figure 7A). The condensed SUVR2 foci are as large as heterochromatin foci that are indicated by DAPI staining and the H3K27me1 mark (Figure 7A). The condensed SUVR2 foci are close to but not colocalized with the heterochromatin foci (Figure 7A), which is consistent with the role of SUVR2 in silencing RdDM target loci rather than genomic loci at heterochromatin regions. When SUVR2 is present as condensed foci in the nucleus, both SUVR1 and CHR19 signals fully overlap with SUVR2 signal (Figure 7B), which supports the interaction of SUVR2 with SUVR1 and CHR19. However, we did not find that the SUVR2 signals overlap with the signals of the RdDM components DRM2, NRPE1, and KTF1 (Supplementary information, Figure S4A-S4C). The data are consistent with the finding that SUVR2 forms a tight complex with SUVR1 and associates with the chromatin-remodeling protein CHR19 (Figure 6A-6H).

### CHR19, CHR27, CHR28, and SUVR1 are required for transcriptional gene silencing

**Figure 8:**
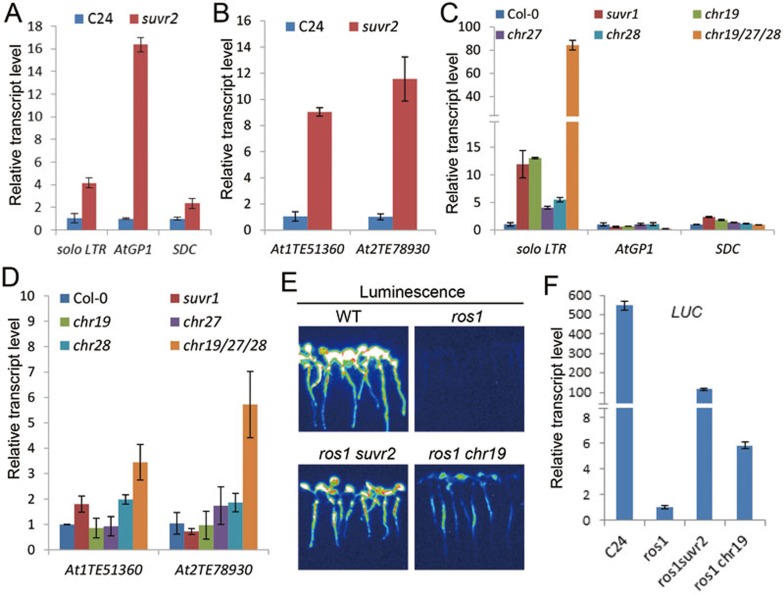
SUVR1 and the SNF2 chromatin-remodeling proteins CHR19, CHR27, and CHR28 are required for transcriptional gene silencing. **(A)** The effect of *suvr2* on the RNA transcript levels of *solo LTR*, *AtGP1*, and *SDC*. The RNA transcript levels of these loci were determined by quantitative RT-PCR. **(B)** The effect of *suvr2* on the RNA transcript levels of two transposable elements *AT2TE51360* and *AT2TE78930*. **(C)** The effect of *suvr1*, *chr19*, *chr27*, and *chr28* on the RNA transcript levels of *solo LTR*, *AtGP1*, and *SDC*. **(D)** The effect of *suvr1*, *chr19*, *chr27*, and *chr28* on the RNA transcript levels of *AT2TE51360* and *AT2TE78930*. **(E)** The effect of *chr19* on the silencing of the *RD29A-LUC* transgene. The *chr19* mutation was introduced into the *ros1* mutant harboring *RD29A-LUC* transgene by crossing. Luminescence imaging of the wild-type plants, and the *ros1*, *ros1suvr2*, and *ros1chr19* mutant plants is shown. **(F)** The expression of the luciferase reporter gene was determined by quantitative RT-PCR in the wild type, *ros1*, *ros1suvr2*, and *ros1chr19* plants harboring the *RD29A-LUC* transgene.

Because SUVR2 associates with the chromatin-remodeling proteins CHR19, CHR27, and CHR28, it is possible that the three chromatin-remodeling proteins function in transcriptional gene silencing as SUVR2 does. Quantitative RT-PCR indicated that the silencing of well-studied RdDM target loci such as *solo LTR*, *AtGP1*, and *SDC* is suppressed in the *suvr2* single mutant (Figure 8A), which is consistent with the observation in the *ros1suvr2* double mutant (Figure 1C). We tested the effect of *suvr2* on transcriptional silencing at several other previously identified RdDM target loci^47,65^. Our results indicated that *AT1TE51360*, *AT2TE78930*, *ERT7*, *ERT12*, and *ERT14* but not *ERT9* are derepressed in the *suvr2* mutant (Figure 8B, Supplementary information, Figure S10A), further confirming that SUVR2 acts in transcriptional silencing at a subset of RdDM target loci. To determine the function of CHR19, CHR27, and CHR28 in transcriptional silencing, we obtained the mutants of *CHR19*, *CHR27*, and *CHR28*, which are Salk_054130C, Salk_063135C, and Salk_057016C, respectively, and generated a *chr19chr27chr28* triple mutant (*chr19/27/28*) by crossing. No morphological phenotype was visualized in the *chr19/27/28* triple mutant and each of the single mutants. Quantitative RT-PCR analysis indicated that the silencing of *solo LTR* and *ERT14* is clearly affected in the *chr19*, *chr27*, and *chr28* single mutants, and the effect is markedly enhanced in the *chr19/27/28* triple mutant (Figure 8C, Supplementary information, Figure S10B). Thus, the function of CHR19, CHR27, and CHR28 in the silencing of *solo LTR* and *ERT14* is at least partially redundant. The silencing of *AT1TE51360*, *AT2TE78930*, *ERT7*, *ERT9*, and *ERT12* is either not affected or slightly affected in the *chr19*, *chr27*, and *chr18* single mutants but is significantly relieved in the *chr19/27/28* triple mutant (Figure 8D, Supplementary information, Figure S10B). The function of CHR19, CHR28, and CHR29 in transcriptional silencing at these loci is clearly redundant. The silencing of *AtGP1* and *SDC* is not markedly affected even in the *chr19/27/28* triple mutant (Figure 8C), suggesting that CHR19/27/28 are not required for the silencing of *AtGP1* and *SDC*.

These results indicate that *solo LTR*, *AT1TE51360*, *AT2TE78930*, *ERT7*, *ERT12*, and *ERT14* are not only targeted by SUVR2 but also by CHR19/27/28, suggesting that CHR19, CHR27, and CHR28 are functionally associated with SUVR2 at these loci. However, *AtGP1* and *SDC* are targeted by SUVR2 but not by CHR19/27/28 (Figure 8A and 8C), whereas *ERT9* is targeted by CHR19/27/28 but not by SUVR2 (Supplementary information, Figures S10A and S9B). These results suggest that SUVR2 and CHR19/27/28 function together at some target loci but have independent roles at other loci.

SUVR2 is required for the silencing of the *RD29A-LUC* transgene in the *ros1* mutant background (Figure 1A and 1B). To determine whether CHR19 is required for the silencing of *RD29A-LUC*, we introduced the *chr19* mutation into the *ros1* mutant by crossing. Luminescence imaging of *ros1chr19* indicated that the *chr19* mutation relieves the silencing of *RD29A-LUC* in *ros1* (Figure 8E), which was confirmed by quantitative RT-PCR (Figure 8F). These results suggest that like SUVR2, CHR19 is involved in the silencing of the *RD29A-LUC* transgene. The effect of *chr19* on the silencing of *RD29A-LUC* is much weaker than that of *suvr2* (Figure 8E and 8F), which is consistent with the observation that CHR19 is functionally redundant with CHR27 and CHR28 in the transcriptional gene silencing of many loci (Figure 8C and 8D, Supplementary information, Figure S10B).

The effect of *suvr1* on the silencing of RdDM target loci was determined by quantitative RT-PCR. The result indicated that *suvr1* affects transcriptional gene silencing at a subset of SUVR2 target loci (Figure 8C and 8D, Supplementary information, Figure S10A and S10B). The involvement of SUVR1 in the silencing of SUVR2 target loci is consistent with the finding that SUVR1 and SUVR2 interact with each other and form a tight complex *in vivo*. To determine whether SUVR1 is required for transgene silencing, we introduced the *suvr1* mutation into the *ros1* mutant harboring the *RD29A-LUC* transgene. The *suvr1* mutation weakly suppresses the silencing of *RD29A-LUC*, and the effect of *suvr1* on transgene silencing is much weaker than that of *suvr2* (Supplementary information, Figure S11). We integrated the *suvr1* mutation into the *ros1suvr2* double mutant and found that in the *ros1suvr1suvr2* mutant, the expression of *RD29A-LUC* is not further enhanced compared to that in the *ros1suvr2* mutant (Supplementary information, Figure S11). These results suggest that SUVR1 and SUVR2 have non-redundant function and both of them are necessary for transcriptional gene silencing.

### The function of SUVR2 and CHR19/27/28 in DNA methylation, siRNA accumulation, and nucleosome positioning

**Figure 9:**
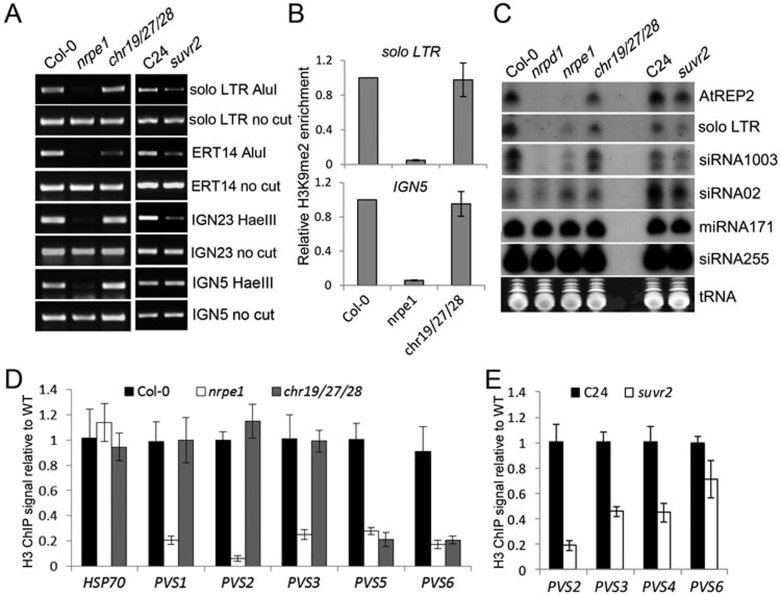
SUVR2 and the SNF2 chromatin-remodeling proteins are involved in DNA methylation, siRNA accumulation, and nucleosome positioning. **(A)** The effect of *suvr2* and *chr19/27/28* on DNA methylation at RdDM target loci. Genomic DNA was digested by the DNA methylation-sensitive restriction enzyme *Alu*I or *Hae*III, followed by PCR. **(B)** The effect of *nrpe1* and *chr19/27/28* on H3K9 dimethylation at RdDM target loci *solo LTR* and *IGN5*. **(C)** The effect of *suvr2* and *chr19/27/28* on the accumulation of Pol IV-dependent siRNAs. Small RNA accumulation was measured by small RNA northern blotting. miRNA171 and ta-siRNA255 were included as controls that are not affected in the Pol IV mutant *nrpd1*. The tRNA image on the ethium bromide-stained gel was shown as a small RNA loading control. **(D**, **E)** The effect of *nrpe1*, *chr19/27/28*, and *suvr2* on Pol V-stabilized nucleosome positioning. The occupancy of nucleosomes on chromatin was determined by MNase digestion followed by H3 ChIP and quantitative PCR. The ChIP signal of *HSP70* was shown as a negative control^47^. ChIP signals were normalized to the actin gene *ACT2*.

The bisulfite sequencing results showed that SUVR2 weakly affects DNA methylation at a subset of RdDM target loci (Figure 2B). Because CHR19, CHR27, and CHR28 associate with SUVR2 and are required for transcriptional gene silencing, we asked whether the function of CHR19, CHR27, and CHR28 in transcriptional gene silencing is related to DNA methylation. The transcript levels of *solo LTR* and *ERT14* are increased in *suvr2* and the *chr19/27/28* triple mutant (Figure 8A and 8C, Supplementary information, Figure S10A and S10B). In the *suvr2* mutant, consistent with the increased expression of *solo LTR* and *ERT14*, the DNA methylation levels at the two loci are weakly reduced (Figure 9A). In the *chr19/27/28* mutant, DNA methylation is reduced for *ERT14* but not for *solo LTR* even though the transcript levels of both *ERT14* and *solo LTR* are increased (Figures 9A and 8C, Supplementary information, Figure S10B). Moreover, we found that the DNA methylation of the RdDM target locus *IGN5* is not affected by *suvr2* and *chr19/27/28*, whereas the DNA methylation of another RdDM target locus *IGN23* is mildly reduced by *suvr2* but not by *chr19/27/28* (Figure 9A). The aforementioned bisulfite sequencing assay indicated that *suvr2* significantly reduces DNA methylation at *solo LTR* and *MEA-ISR* sites (Figure 2B). We performed bisulfite sequencing to determine the effect of *chr19/27/28* on DNA methylation at these two sites and found that their DNA methylation levels are not significantly affected in the *chr19/27/28* mutant (Supplementary information, Figure S12A and S12B). *5S rDNA* methylation was reduced by *suvr2* at CHH sites and the effect of *suvr2* is weaker than that of *nrpe1* (Supplementary information, Figure S12C). *5S rDNA* methylation is slightly reduced by *chr19/27/28* at CHH sites but the effect is even weaker than that of *suvr2* (Supplementary information, Figure S12C). These results suggest that CHR19/27/28 affect DNA methylation to a lesser extent than SUVR2 and may have DNA methylation-independent roles in transcriptional gene silencing.

Previous reports suggest that RdDM components are required for H3K9 dimethylation at RdDM target loci^11,57,58^. We performed ChIP-PCR to test whether CHR19/27/28 are required for H3K9 dimethylation (Figure 9B). Our result indicated that the H3K9me2 levels at *solo LTR* and *IGN5* are drastically decreased in the Pol V mutant *nrpe1* (Figure 9B), which is consistent with previous reports^11,57^. However, the H3K9me2 ChIP assay indicated that SUVR2 is not required for H3K9me2 at its target loci including the transgenic and endogenous *RD29A* promoters, *solo LTR*, and *IGN5* (Figure 5A-5D). Here, we found that CHR19/27/28 is dispensable for H3K9me2 at *solo LTR* and *IGN5* (Figure 9B), which is consistent with the action of SUVR2 at these loci. Thus, the involvement of SUVR2 and CHR19/28/29 in transcriptional silencing is mostly likely through an as yet unknown mechanism.

The function of SUVR2 and CHR19/27/28 in small RNA accumulation was examined by northern blotting (Figure 9C). The accumulation of Pol IV- and Pol V-dependent siRNAs including *AtREP2* siRNA, *solo LTR* siRNA, and siRNA1003 is slightly decreased in *suvr2* (Figure 9C). The accumulation of miRNA171 and trans-acting siRNA255 is not affected by *suvr2* (Figure 9C), suggesting that the function of SUVR2 is specifically associated with Pol IV-dependent siRNAs but not with other types of small RNAs. However, the effect of *suvr2* on siRNA accumulation is much weaker than that of the Pol V mutation *nrpe1* (Figure 9C). The effect of *suvr2* on Pol IV- and Pol V-dependent siRNAs as determined by northern blotting is consistent with our small RNA deep sequencing data (Figure 2C and 2D, Supplementary information, Table S1). In the *chr19/27/28* mutant, the accumulation of *AtREP2* siRNA, *solo LTR* siRNA, and siRNA1003 is weakly decreased (Figure 9C). The effect of *chr19/27/28* on siRNA accumulation is similar to that of *suvr2*. Moreover, neither *suvr2* nor *chr19/27/28* affects the accumulation of siRNA02, which shows a Pol IV-dependent and Pol V-independent pattern (Figure 9C). Thus, *suvr2* and *chr19/27/28* may indirectly affect the accumulation of Pol IV- and Pol V-dependent siRNAs at RdDM target loci.

As CHR19/27/28 are putative ATP-dependent SNF2 chromatin-remodeling proteins, it is possible that the involvement of CHR19/27/28 in transcriptional gene silencing is correlated with their function in nucleosome positioning. Pol V-stabilized nucleosomes were previously identified by MNase (Microccocal Nuclease) digestion followed by DNA deep sequencing^47^. In the Pol V mutant *nrpe1*, these nucleosomes were destabilized and their levels decreased^47^. We performed MNase digestion followed by H3 ChIP-PCR to determine whether *chr19/27/28* may affect the occupancy of these nucleosomes on chromatin (Figure 9D). The nucleosome occupancy at *PVS1*, *PVS2*, *PVS3*, *PVS5*, and *PVS6* sites was markedly decreased in *nrpe1* (Figure 9D), which is consistent with the previous report^47^. In *chr19/27/28*, the nucleosome occupancy was reduced at the *PVS5* and *PVS6* sites but not at the *PVS1*, *PVS2*, and *PVS3* sites (Figure 9D), suggesting that CHR19/27/28 are required for the occupancy of a subset of Pol V-stabilized nucleosomes on chromatin. The *suvr2* mutant used in this study is in the C24 ecotype, in which the occupancy of Pol V-stabilized nucleosomes is detectable at *PVS2*, *PVS3*, *PVS4*, and *PVS6* (Figure 9E). In the *suvr2* mutant, the occupancy of Pol V-stabilized nucleosomes is reduced at all these loci (Figure 9E). These results are consistent with the finding that CHR19/27/28 are required for transcriptional gene silencing at a subset of SUVR2 target loci (Figure 8A-8D). Based on a previous report^47^, the SWI/SNF chromatin-remodeling complex component Brahma (BRM) affects the same subset of the nucleosome sites, suggesting that CHR19/27/28 and BRM may be recruited to specific chromatin target loci by the same mechanism. Pol V-produced noncoding RNAs are thought to recruit IDN2 and then the SWI/SNF complex to chromatin, thereby facilitating nucleosome positioning^47^. Pol V-produced noncoding RNAs are probably not only required for the recruitment of the SWI/SNF complex but also for the recruitment of CHR19/27/28.

## Discussion

SUVR2 is a member of the SU(VAR)3-9-related protein family in *Arabidopsis*. In this family, SUVR4 is an active histone methyltransferase and is involved in histone H3K9 methylation and transcriptional gene silencing^28^, but the histone methyltransferase activity of SUVR4 is much weaker than that of the human SUV39H1 as determined by an *in vitro* assay^28^. SUVR1, SUVR2, and SUVR5 have no histone methyltransferase activity as determined by *in vitro* assays (Figure 5G)^28,30^, even though SUVR5 is involved in histone H3K9 methylation *in vivo*^28,30^. The SET domain of SUVR2 is conserved in the SU(VAR)3-9 homologs including Dim-5, Clr4, and G9a (Supplementary information, Figure S6B). Previous structural and biochemical studies suggest that some conserved residues in the SET domains of the SU(VAR)3-9 homologs are directly responsible for the binding of the substrate histone H3 and the methyl group donor SAM^20,64^. These residues are critical for the catalytic activity of the histone methyltransferases. In the SET domain of SUVR2, substitutions are present in some of these critical residues, which include the H3K9-binding sites corresponding to N247 and F281 of Dim-5, as well as the SAM-binding sites corresponding to R155 and Y204 of Dim-5 (Supplementary information, Figure S6B). These substitutions are likely responsible for the loss of the SUVR2 histone methyltransferase activity. Our study here showed that mutations of the conserved catalytic residues in the SET domain of SUVR2 do not affect the function of SUVR2 in transcriptional gene silencing (Figure 5E-5F), which is consistent with the observation that SUVR2 has no histone methyltransferase activity as determined by the *in vitro* assay. In *Arabidopsis*, the three SU(VAR)3-9 homologs SUVH4/KYP, SUVH5, and SUVH6 have histone H3K9 methyltransferase activity as determined by *in vitro* and *in vivo* assays^21,22,25^. The three active H3K9 methyltransferases contain a conserved SRA domain that can directly bind to both symmetric and asymmetric cytosine sites^24,25^. Histone H3K9 dimethylation at RdDM target loci is likely catalyzed by SUVH4/KYP, SUVH5, and SUVH6 in *Arabidopsis*.

The WILYD domain was previously identified as the domain required for ubiquitin binding in SUVR4^29^. The domain is thought to be responsible for conversion of the enzyme activity of SUVR4 from H3K9 dimethyltransferase to trimethyltransferase. Unlike SUVR4, no histone methyltransferase activity was detected for SUVR2 even when ubiquitin was added (Figure 5G), suggesting that the WILYD domain of SUVR2 may have a different role compared to that of SUVR4. We found that mutations of the conserved residues in the WILYD domain do not affect the function of SUVR2 in transcriptional gene silencing. Thus, the ubiquitin-binding ability of the WILYD domain is dispensable for the function of SUVR2 in transcriptional gene silencing.

SUVR2 was recently reported as a canonical RdDM component^5^. We found that DNA methylation and transcriptional silencing is affected by *suvr2* at a subset of RdDM target loci, which is consistent with a role of SUVR2 in the RdDM pathway. Although *suvr2* significantly affects transcriptional gene silencing at RdDM target loci, DNA methylation is either not affected or weakly affected in the *suvr2* mutant (Figure 2A and 2B). These results suggest that SUVR2 may also function downstream of the RdDM pathway or may have an RdDM-independent role in transcriptional gene silencing.

Our observation that *suvr2* shows synergistic effects with canonical RdDM mutations on the silencing of a subset of RdDM target loci (Figure 3B) supports that SUVR2 functions in transcriptional gene silencing at least partially independently of RdDM. Our results suggest that SUVR2 functions not only at a subset of RdDM target loci but also at loci that are not targeted by RdDM (Figure 4A-4F, Supplementary information, Tables S3,S4,S5,S6). Based on our quantitative RT-PCR results (Figure 3B), common target loci shared by SUVR2 and RdDM can be divided into two classes. Class I loci are represented by *solo LTR* and *AtGP1*. These loci are not further activated by *suvr2* when the RdDM pathway is completely disrupted in the *nrpd1* and *nrpe1* mutant backgrounds. The existence of class I loci suggests that SUVR2 and RdDM act non-redundantly in transcriptional gene silencing. At these loci, SUVR2 probably acts downstream of the RdDM pathway and is required for the transduction of DNA methylation to transcriptional repression. Class II loci are represented by *SDC* and *ERT7* (Figure 3B). The transcription of class II loci is synergistically activated when *suvr2* is combined with *nrpd1* or *nrpe1*. The synergistic effect between *suvr2* and canonical RdDM mutations indicates that the function of SUVR2 is independent of the RdDM pathway at class II loci. These results suggest that the cooperation between SUVR2 and RdDM is more complex than expected. Some specific chromatin features in class I and class II loci may determine how SUVR2 cooperates with RdDM.

Previous reports indicate that the RdDM pathway cooperates with several other chromatin silencing regulators including DDM1, HAD6, MOM1, and MORC6/DMS11^26,34,39,41,42,65^. These results suggest that a stable state of chromatin silencing is dependent on the interplay of multiple transcriptional silencing mechanisms. It is interesting to understand when and how SUVR2 mediates transcriptional gene silencing in DNA methylation-dependent and -independent manners. The association of SUVR2 with the chromatin-remodeling proteins CHR19, CHR27, and CHR28 provides a possible functional mechanism for SUVR2 in transcriptional gene silencing. Several chromatin-remodeling proteins were previously identified as regulators of transcriptional gene silencing^31,32,33,34,35^. The chromatin-remodeling proteins DRD1 and CLSY1 act as RdDM regulators^32,33^. DRD1 interacts with DMS3 and RDM1, forming a DDR complex required for the occupancy of Pol V on chromatin and for Pol V transcription^11,15,16^. CLSY1 is required for proper localization of NRPD1 and RDR2 in the nucleus^33^. DDM1 facilitates the access of DNA methyltransferases to H1-containing heterochromatin and is required for maintenance of DNA methylation^34^. These results suggest that chromatin remodeling mediated by DRD1, CLSY1, and DDM1 contributes to DNA methylation and transcriptional gene silencing. We demonstrate that the involvement of SUVR2 and CHR19/27/28 in transcriptional gene silencing is not always correlated with DNA methylation. Pol V-stabilized nucleosomes were thought to participate in transcriptional gene silencing^47^. We found that SUVR2 is required for Pol V-stabilized nucleosome positioning on chromatin, whereas CHR19/27/28 are responsible for the occupancy of a subset of these nucleosomes (Figure 9D and 9E). Previous studies suggest that nucleosome positioning is related to both DNA methylation and Pol II transcription^66,67^. The involvement of SUVR2 and CHR19/27/28 in nucleosome positioning provides a possible DNA methylation-independent mechanism underlying transcriptional gene silencing.

SUVR2 is required for nucleosome positioning at all tested Pol V-stabilized nucleosome loci, whereas CHR19/27/28 are only responsible for nucleosome positioning at a subset of these loci (Figure 9D and 9E). A previous report indicated that a SWI/SNF chromatin-remodeling complex is required for nucleosome positioning for a subset of Pol V-stabilized nucleosome loci^47^. We found that CHR19/27/28 are involved in nucleosome positioning at the same subset of Pol V-stabilized nucleosomes as the SWI/SNF complex (Figure 9D). The recruitment of the SWI/SNF complex to chromatin was thought to be guided by Pol V-produced noncoding RNAs and IDN2^47^. CHR19/27/CHR28 may be recruited to chromatin in a similar manner to the SWI/SNF complex and are involved in nucleosome positioning at a subset of RdDM target loci. We demonstrated that SUVR2 directly binds to histone H3 *in vitro* (Figure 5H). The binding of SUVR2 to histone H3 may be necessary for the interaction of SUVR2 to chromatin. Thus, in addition to Pol V-produced nocoding RNAs and IDN2, SUVR2 is probably required for the recruitment of CHR19/27/28 to chromatin. The recruited chromatin-remodeling proteins then mediate nucleosome positioning, thereby contributing to DNA methylation or directly repressing Pol II transcription.

Our results demonstrated that SUVR2 and CHR19/27/28 weakly affect the accumulation of Pol IV- and Pol V-dependent 24-nt siRNAs (Figures 2C, 2D and 9C). Pol V is responsible for producing scaffold noncoding RNAs and acts downstream of siRNA biogenesis^11^. However, in the RdDM pathway, Pol V is also involved in accumulation of a subset of Pol IV-dependent siRNAs^68^. The effect of Pol V on siRNA accumulation was thought to depend on its function in DNA methylation and transcriptional gene silencing^68,69^. Similarly, SUVR2 and CHR19/27/28 may affect siRNA accumulation through a self-reinforcing loop between siRNA accumulation and transcriptional gene silencing at RdDM target loci.

The function of SUVR2 in transcriptional gene silencing is not always dependent on the chromatin-remodeling proteins CHR19/27/28 (Figure 8A-8D). Although SUVR2 associates with CHR19/27/28, the size of the main SUVR2 complex is much higher than that of the main CHR19 complex (Figure 6H), suggesting that SUVR2 forms a separate complex without CHR19. The fractions of the SUVR2 complex largely overlap with those of the SUVR1 complex (Figure 6H). We propose that SUVR2 not only associates with CHR19/27/28 but also forms a heteromer with SUVR1, thereby facilitating DNA methylation and transcriptional gene silencing. Further studies are required to clarify how the SUVR1/SUVR2 heteromer participates in transcriptional gene silencing in a CHR19/27/28-independent manner.

## Materials and Methods

### Plant materials, map-based cloning, and complementation testing

*Arabidopsis* seedlings were grown on MS medium for ten days and were then subject to luminescence imaging after cold treatment at 4 °C for 4 days. For kanamycin resistance detection, seedlings were grown on 150 mM kanamycin MS medium and were imaged after two weeks. For map-based cloning, the *ros1#87* double mutant in the C24 ecotype was crossed to the T-DNA insertion line of the *ros1* mutant in the Col-0 ecotype (Salk_045303), and the F2 generation was used for mapping. The bright ones of F2 were selected for DNA extraction followed by PCR of SSLP (Simple sequence length polymorphism) markers. The mutation was mapped to a 974 kb interval on the chromosome 5. The *ros1#87* genome was sequenced, and a G-to-A mutation was found in *SUVR2* at the junction of the second intron and the third exon. The full genomic sequence of *SUVR2* with its own promoter was cloned into the vector modified from pCAMBIA1305 to have a 3× Flag tag at its 3′-end. This construct was transformed into *ros1#87* for complementation assay. For the point mutation rescue test, the genomic sequence of *SUVR2* with each indicated mutation was cloned and transformed into *ros1#87* as well. The *ros1suvr2* double mutant was crossed with the wild-type and the *suvr1* mutant (Salk_012786C) to obtain *suvr2*, *ros1suvr1*, and *ros1suvr1suvr2*. Moreover, we crossed *ros1suvr2* with *ros1nrpd1*, *ros1nrpe1*, and *ros1dcl3* to obtain *ros1nrpd1suvr2*, *ros1nrpe1suvr2*, and *ros1dcl3suvr2*, respectively. The *chr19*, *chr27*, and *chr28* single mutants were used to generate the *chr19chr27chr28* triple mutant by crossing. The *chr19* mutant was crossed with the *ros1* mutant harboring the *RD29A-LUC* transgene to obtain the *ros1chr19* double mutant.

### DNA methylation assay

DNA methylation was determined by bisulfite sequencing, Southern blotting, and chop-PCR. For bisulfite sequencing, genomic DNA from 2-week-old seedlings was extracted and 2 μg of genomic DNA was treated by the sodium bisulfite reagent (Qiagen, 59104) so that unmethylated cytosines were converted to uracils. The converted DNA was purified and used for PCR with primers indicated in Supplementary information, Table S8. The PCR products were cloned into T-vector for sequencing. For each sample, more than 15 clones were sequenced, and the percentage of CG, CHG and CHH methylation was analyzed separately online by CyMate website^70^. For Southern blotting, 5 μg of genomic DNA was digested with the DNA methylation-sensitive restriction enzymes *Hpa*II, *Msp*I, and *Hae*III. The digested DNA was subjected to Southern blotting for *5S rDNA*. For chop-PCR, genomic DNA was digested with the DNA methylation sensitive restriction enzymes *Hae*III and *Alu*I overnight, followed by amplification of *ERT14*, *solo LTR*, *IGN5*, and *IGN23*.

### RNA analysis and small RNA northern blotting

For quantitative RT-PCR, total RNA was extracted using Trizol reagent, followed by reverse transcription and PCR (TaKaRa, RR012A). For the detection of Pol V-produced transcripts, one-step RT-PCR was performed as described previously^11^. For RNA-seq analysis, total RNA was isolated from two-week-old seedlings and used for generating RNA libraries. The RNA libraries were subjected to single-end sequencing by HiSeq 2000 (Illumina). RNA-seq data were analyzed as described before^71^.

For small RNA northern blotting, small RNA was extracted from two-week-old seedlings with Trizol reagent, and run on a 15% polyacrylamide gel with the method described^49^. The small RNAs were transferred to Hybond N^+^ membrane electrically (Amersham, RPN3050N), and then subjected to small RNA hybridization. Two types of probes were used: DNA oligonucleotides or PCR products. DNA oligonucleotides were labeled with γ-^32^P-ATP, and PCR products with α-^32^P-dCTP. The membrane was incubated in PerfectHyb buffer (Sigma, H7033) overnight at 38 °C for hybridization.

### Affinity purification and mass spectrometry

Six grams of flower tissue from the *SUVR2-Flag* transgenic plants as well as the wild-type control were used to prepare protein extracts as previously described^8^. Anti-Flag M1 agarose (Sigma, A4596) was incubated with the protein extracts and washed. The agarose-bound proteins were eluted with 3× Flag peptides (Sigma) and run on SDS-PAGE followed by silver staining (Sigma, PROT-SIL1). The silver-stained proteins were de-stained and digested in-gel with trypsin (10 ng/μl trypsin, 50 mM ammonium bicarbonate, pH 8.0) at 37 °C overnight. The digested peptides were purified for mass spectrometry as previously reported^44^.

### Immunolocalization

Nuclei extracted from rosette leaves were fixed in 4% formaldehyde and then applied to slides as previously described^8^. The slides were blocked in PBS buffer with 3% BSA, followed by primary antibody hybridization. Secondary anti-mouse antibody TRITC (Tetramethyl Rhodamine Isothiocyanate-conjugated) (Invitrogen, Z25005) and anti-rabbit antibody FITC (Fluorescein Isothiocyanate-conjugated) (Invitrogen, 710369) were added after washing of slides, and incubated on slides at 37 °C. Chromatin was counterstained with DAPI (4′-6-Diamidino-2-phenylindole). Images were collected by SPINNING DISK confocal microscopy. The antibodies for H3K9me1, H3K9me2, H3K9me3, H3K27me1, H3K27me2, and H3K27me3 were from Millipore.

### Coimmunoprecipitation

*SUVR2-Flag* and *SUVR2-Myc* were constructed in the modified pCAMBIA1305 vector and introduced into *Arabidopsis*. *SUVR2-Myc* transgenic plants were crossed to the plants harboring *SUVR1-Flag*, *SUVR2-Flag*, *NRPE1-Flag*, *KTF1-Flag*, or *DRM2-Flag* transgene, whereas *SUVR2-Flag* transgenic plants were crossed to the *CHR19-Myc* or *RDM1-Myc* transgenic plants to generate the offspring plants harboring two tagged proteins. Protein extracts were isolated from the plants and incubated with anti-c-Myc agarose (Sigma, A7470), anti-Flag M1 agarose (Sigma, A 4596), and anti-AGO4 conjugated agarose (Agrisera, AS09617). After the agarose-bound proteins were washed, the proteins were boiled and run on SDS-PAGE for western blotting.

### Histone methyltransferase activity assay

The full-length *SUVR2* and the SET domain of *SUVR2* were cloned in frame with 6× His in the pET28a vector, and expressed in bacteria. The histone methyltransferase activity assay was carried out according to the method previously described^29^. The human SU(VAR)3-9 homolog G9a was served as a positive control.

### Chromatin immunoprecipitation

H3K9me2 and H3K9me3 ChIP assays were carried out following the procedure previously described^11^. Two-week-old seedlings were fixed in 1% formaldehyde followed by washing for 5 times. Nuclei were extracted from the material and incubated with H3K9me2 antibody (abcam, ab1220) or H3K9me3 antibody (Millipore, 17-625) at 4 °C overnight. Chromatin bound by H3K9me2 or H3K9me3 was purified and used for PCR with sequence-specific primers listed in Supplementary information, Table S8. The occupancy of H3K9me2 or H3K9me3 on the actin gene *ACT2* was used as a negative control. We determined nucleosome positioning according to the method described previously^47^. Briefly, the nuclear extract was digested with microccocal nuclease (MNase, NEB, #M0247S) followed by H3 ChIP (abcam, ab1791) and quantitative PCR.

### Yeast two-hybrid assay

The interaction of SUVR2 with either the full-length or truncated SUVR2 was determined by yeast two-hybrid assay. The full-length and truncated versions of SUVR2 were constructed in the pGBKT7 plasmid, whereas the full-length SUVR2 was constructed in the pGADT7 plasmid. The pGBKT7 and pGADT7 plasmids were cotransformed into the yeast strain. The transformed yeast cells were grown on SD-TL (the synthetic dropout medium minus Trp and Leu) and the positive colonies were used for yeast two-hybrid. All the yeast strains harboring pGADT7 and pGBKT7 constructs were grown on SD-TLH (the medium minus Trp, Leu, and His) supplemented with 20 mM 3-AT as well as on SD-TL. The growth on SD-TLH indicates the expression of the His synthesis gene, suggesting the interaction of the proteins in the pGADT7 and pGBKT7 plasmids.

## Figures and Tables

**Table 1:** Mass spectrometric analyses of SUVR2, SUVR1, CHR19, and CHR27 affinity purification

AGI code	Protein	SUVR2-Flag		SUVR1-Flag		CHR19-Myc		CHR27-Myc	
		Mascot score	Unique peptides	Mascot score	Unique peptides	Mascot score	Unique peptides	Mascot score	Unique peptides
AT5G43990	SUVR2	18282	46	1232	11	217	3	282	3
AT1G04050	SUVR1	814	10	5199	34	0	0	0	0
AT2G02090	CHR19/ETL1	804	11	0	0	8937	57	0	0
AT3G20010	CHR27	285	5	0	0	0	0	2716	35
AT1G50410	CHR28	98	3	0	0	0	0	0	0

## Supplementary Information

Supplementary information, Figure S1 Map-based cloning and complementation testing of *SUVR2*.

Supplementary information, Figure S2 The DNA methylation level of *IGN23* in the *SUVR2* transgenic lines in the *ros1suvr2* mutant background.

Supplementary information, Figure S3 SUVR2 does not interact with major RdDM components by co-IP.

Supplementary information, Figure S4 SUVR2 does not colocalize with the RdDM components DRM2, NRPE1, and KTF1 in the nucleus.

Supplementary information, Figure S5 H3K9me3 enrichment is not affected by *nrpe1* and *suvr2*.

Supplementary information, Figure S6 Alignment of SUVR2 and its related sequences.

Supplementary information, Figure S7 Point mutations in the WIYLD domain and the SET domain of SUVR2 have no effect on transcriptional gene silencing.

Supplementary information, Figure S8 The interaction of SUVR2 with SUVR2 was not detected by *in vitro* pull down assays.

Supplementary information, Figure S9 The full-length SUVR2 and truncated SUVR2 versions have no self-activation activity.

Supplementary information, Figure S10 The function of SUVR1, SUVR2, and the chromatin-remodeling proteins in the silencing of the RdDM target loci *ETR7*, *ETR9*, *ETR12*, and *ETR14*.

Supplementary information, Figure S11 SUVR1 and SUVR2 have a redundant function in the silencing of the *RD29A-LUC* transgene.

Supplementary information, Figure S12 The effect of *suvr2* and *chr19/27/28* on DNA methylation.

Supplementary information, Table S1 Numbers of Pol IV-dependent siRNA reads in 500-bp genomic windows for each ecotype.

Supplementary information, Table S2 Summary of reads from RNA-seq.

Supplementary information, Table S3 Differentially expressed genes between *ros1* and *ros1suvr2* identified by RNA-seq.

Supplementary information, Table S4 Differentially expressed genes between *ros1* and *ros1nrpe1* identified by RNA-seq.

Supplementary information, Table S5 Differentially expressed TEs between *ros1* and *ros1suvr2* identified by RNA-seq.

Supplementary information, Table S6 Differentially expressed TEs between *ros1* and *ros1nrpe1* identified by RNA-seq.

Supplementary information, Table S7 Full lists of co-purified proteins identified by mass spectrometric analyses.

Supplementary information, Table S8 List of DNA oligonucleotides used in this study.
