# Supplementary material for: SUVR2 is involved in transcriptional gene silencing by associating with SNF2-related chromatin-remodeling proteins in Arabidopsis
Source: Cell Res. 2014 Nov 25;24(12):1445–65. doi: 10.1038/cr.2014.156 (PMC4260354; doi:10.1038/cr.2014.156)
Supplement: Supplementary information, Figure S1 — Map-based cloning and complementation testing of SUVR2. [file cr2014156x1.pdf]

Fig S1

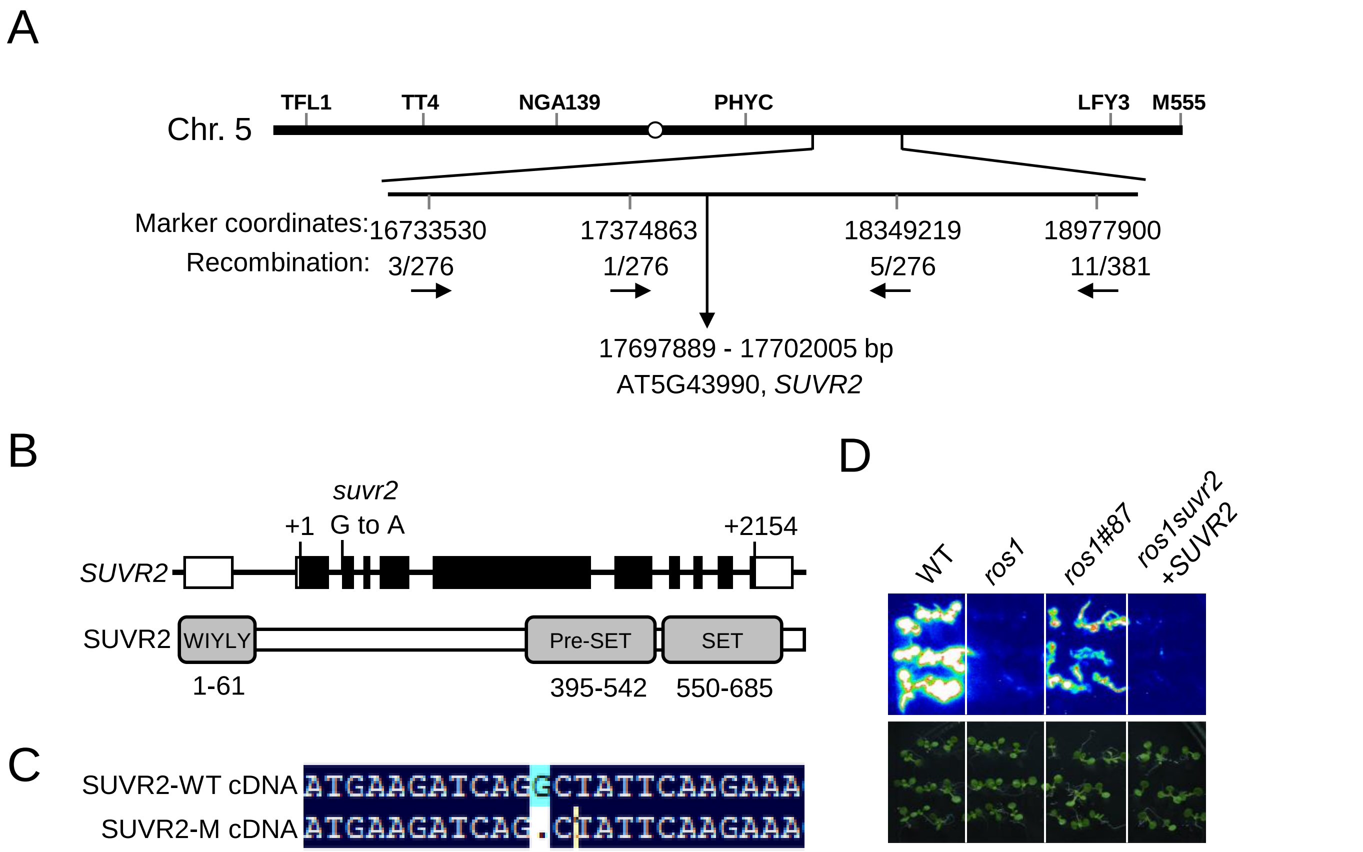

Supplemental Figure S1. Map-based cloning and complementation testing of *SUVR2*. (A) Diagram of the chromosome region that was localized by map-based cloning. The ~1 M region on Chromosome 5 contains a mutation of *SUVR2* in *ros1#87*. (B) Diagrams of the *SUVR2* gene and its encoded protein. The mutation of *SUVR2* in *ros1#87* disrupts the splicing receptor site in the second intron of *SUVR2*. In the SUVR2 protein sequence, the positions of the conserved domains are shown. (C) Alignement of the SUVR2 cDNA sequences from the wild-type and #87 mutant plants. The G to A mutation in #87 leads to one-nucleotide deletion and causes frame shift. (D) The construct harboring the genomic *SUVR2* sequence was transformed into ros1suvr2 for complementation testing. The luminescence images of the *SUVR2* transgenic plants and the control plants are shown.
