# Supplementary material for: SUVR2 is involved in transcriptional gene silencing by associating with SNF2-related chromatin-remodeling proteins in Arabidopsis
Source: Cell Res. 2014 Nov 25;24(12):1445–65. doi: 10.1038/cr.2014.156 (PMC4260354; doi:10.1038/cr.2014.156)
Supplement: Supplementary information, Figure S2 — The DNA methylation level of IGN23 in the SUVR2 transgenic lines in the ros1suvr2 mutant background. [file cr2014156x2.pdf]

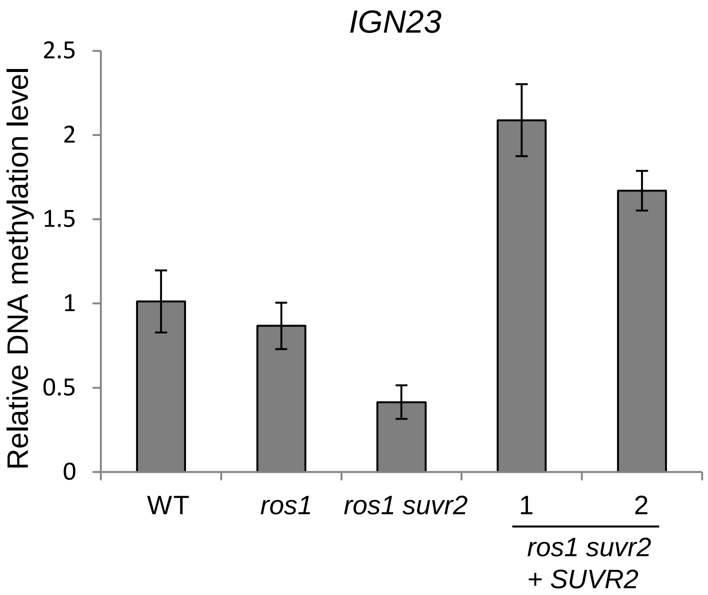

Supplemental Figure S2. The DNA methylation level of *IGN23* in the *SUVR2* transgenic lines in the *ros1suvr2* mutant background. A *SUVR2* construct was transformed into the *ros1suvr2* mutant and two SUVR2 overexpression lines were selected for testing the DNA methylation level of the RdDM target locus *IGN23*. For DNA methylation analysis, genomic DNA was cleaved by the DNA methylation-sensitive restriction enzyme HaeIII, followed by quantitative PCR.
