# Supplementary material for: SUVR2 is involved in transcriptional gene silencing by associating with SNF2-related chromatin-remodeling proteins in Arabidopsis
Source: Cell Res. 2014 Nov 25;24(12):1445–65. doi: 10.1038/cr.2014.156 (PMC4260354; doi:10.1038/cr.2014.156)
Supplement: Supplementary information, Figure S3 — SUVR2 does not interact with major RdDM components by co-IP. [file cr2014156x3.pdf]

Fig S3

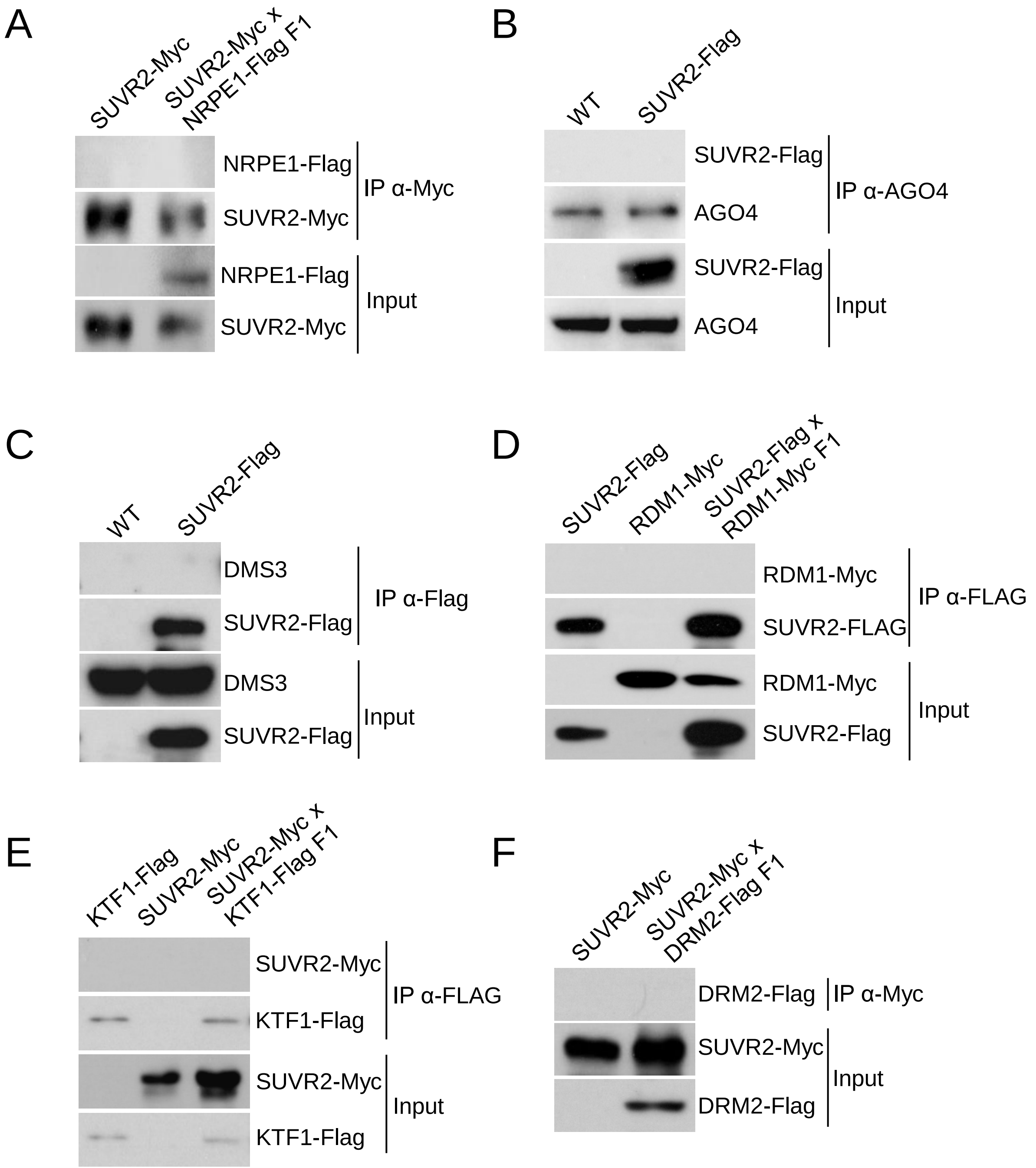

Supplemental Figure S3. SUVR2 does not interact with major RdDM components by co-IP. (A) The interaction between SUVR2 and NRPE1 was tested in the F1 hybrids plants from the cross of *SUVR2-Myc* and *NRPE1-Flag* transgenic plants. (B) The interaction between SUVR2 and AGO4 was tested by co-IP in the *SUVR2-Flag* transgenic plants. (C) The interaction between SUVR2 and DMS3. (D) The interaction between SUVR2 and RDM1. (E) The interaction between SUVR2 and KTF1. (F) The interaction between SUVR2 and DRM2.
