# Supplementary material for: SUVR2 is involved in transcriptional gene silencing by associating with SNF2-related chromatin-remodeling proteins in Arabidopsis
Source: Cell Res. 2014 Nov 25;24(12):1445–65. doi: 10.1038/cr.2014.156 (PMC4260354; doi:10.1038/cr.2014.156)
Supplement: Supplementary information, Figure S4 — SUVR2 does not colocalize with the RdDM components DRM2, NRPE1, and KTF1 in the nucleus. [file cr2014156x4.pdf]

Fig S4

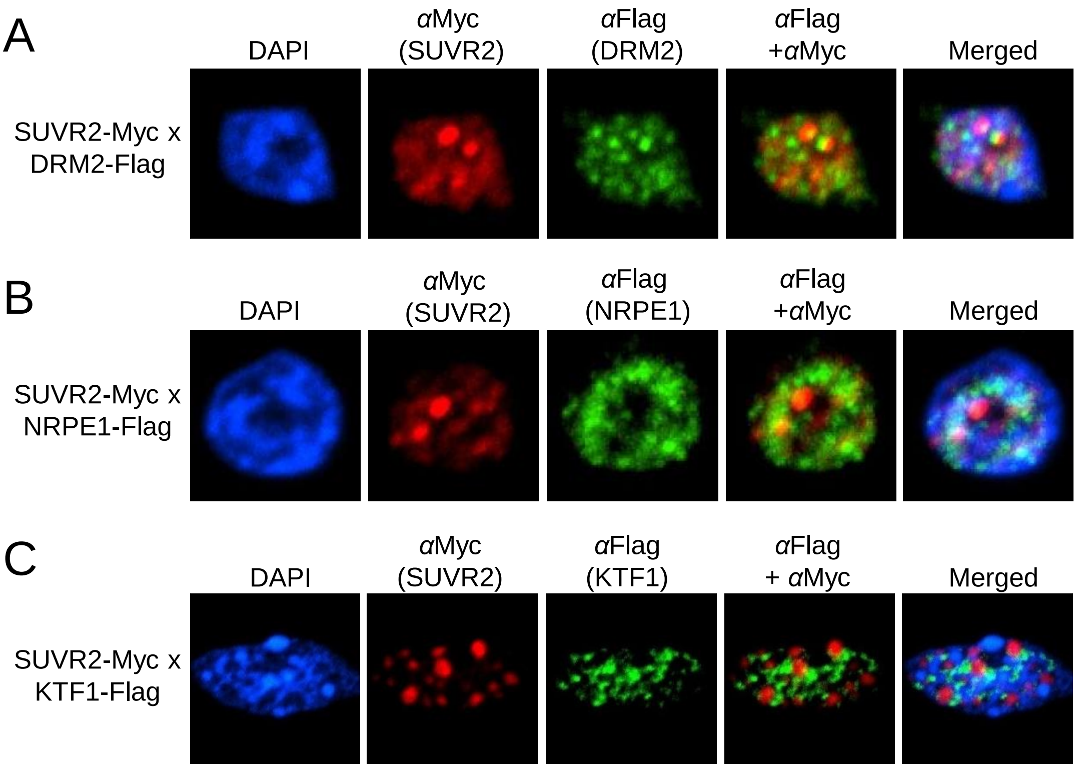

Supplemental Figure S4. SUVR2 does not colocalize with the RdDM components DRM2, NRPE1, and KTF1 in the nucleus. (A, B, C) Immunolocalization was performed to determine whether SUVR2 is colocalized with the RdDM components DRM2, NRPE1, and KTF1. *SUVR2-Myc* transgenic plants were crossed to *DRM2-Flag*, *NRPE1-Flag*, or *KTF1-Flag* transgenic plants. The offspring plants harboring both tagged proteins were used for immunostaining with both anti-Myc antibody and anti-Flag antibody.
