# Supplementary material for: SUVR2 is involved in transcriptional gene silencing by associating with SNF2-related chromatin-remodeling proteins in Arabidopsis
Source: Cell Res. 2014 Nov 25;24(12):1445–65. doi: 10.1038/cr.2014.156 (PMC4260354; doi:10.1038/cr.2014.156)
Supplement: Supplementary information, Figure S5 — H3K9me3 enrichment is not affected by nrpe1 and suvr2. [file cr2014156x5.pdf]

Fig S5

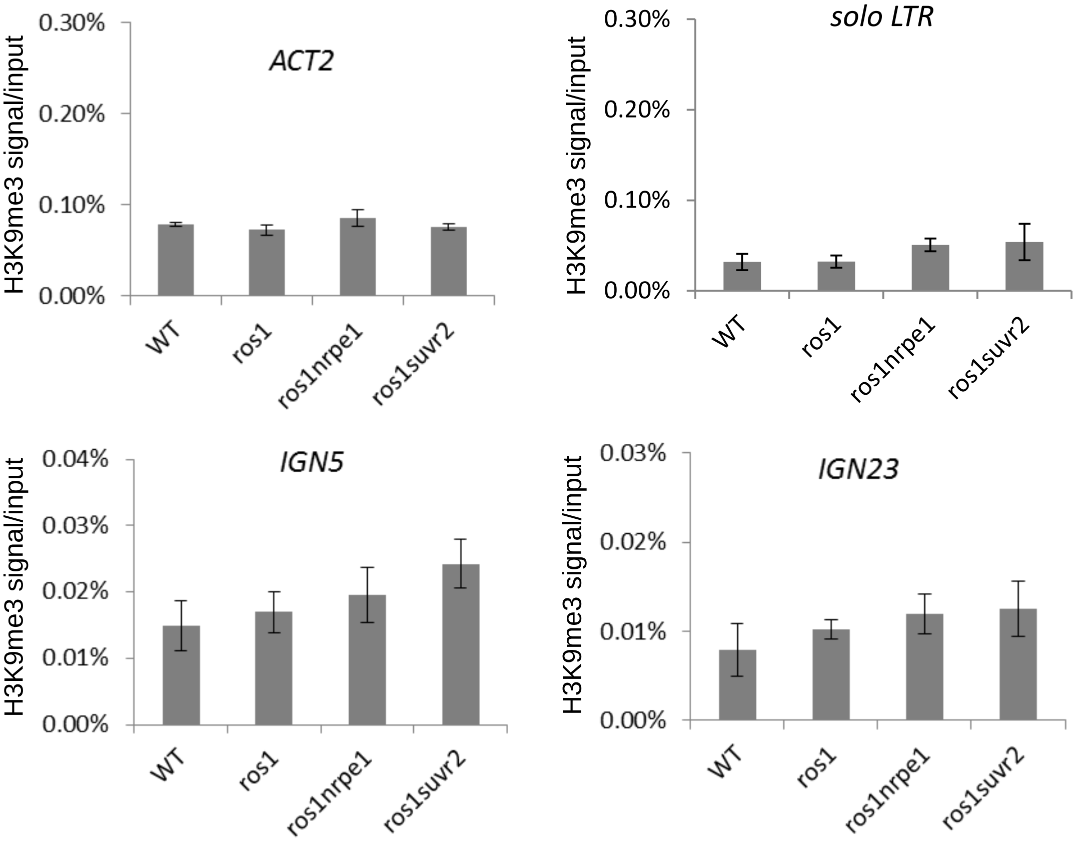

Supplemental Figure S5. H3K9me3 enrichment is not affected by *nrpe1* and *suvr2*. A ChIP-PCR assay was performed to determined H3K9me3 levels in wild type, *ros1*, *ros1nrpe1*, and *ros1suvr2.* H3K9me3 signals were normalized relative to input. The H3K9me3 levels of the RdDM target loci *solo LTR*, *IGN5*, and *IGN23* was determined. *ACT2* was used as a control.
