# Supplementary material for: SUVR2 is involved in transcriptional gene silencing by associating with SNF2-related chromatin-remodeling proteins in Arabidopsis
Source: Cell Res. 2014 Nov 25;24(12):1445–65. doi: 10.1038/cr.2014.156 (PMC4260354; doi:10.1038/cr.2014.156)
Supplement: Supplementary information, Figure S6 — Alignment of SUVR2 and its related sequences. [file cr2014156x6.pdf]

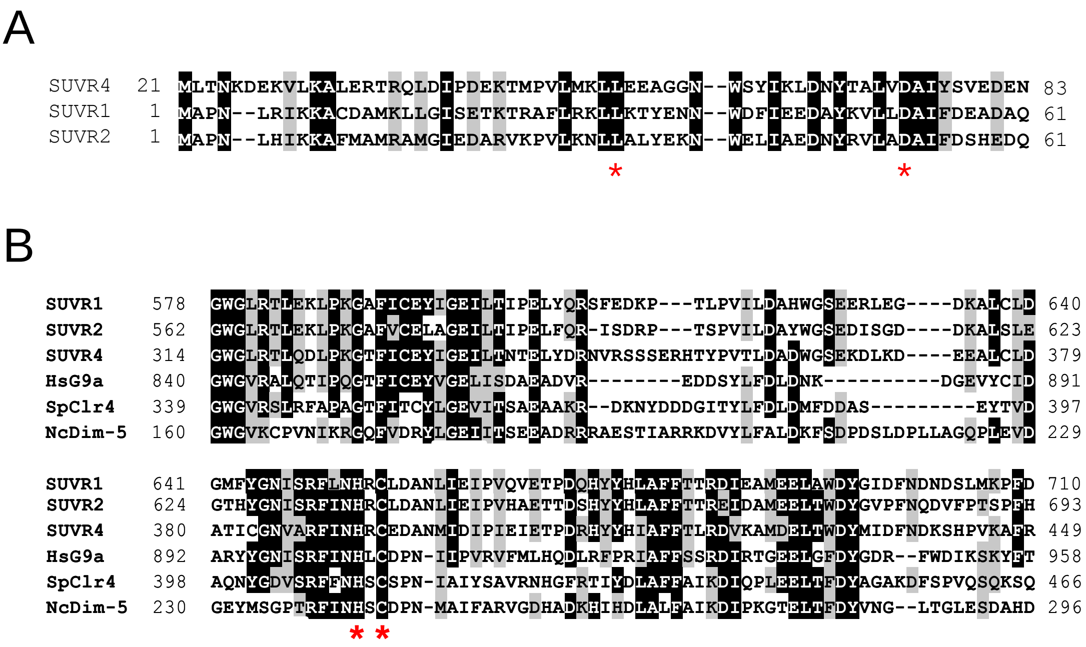

Supplemental Figure S6. Alignment of SUVR2 and its related sequences. The conserved residues that are labeled with stars were mutated in SUVR2 and used for complementation testing in this study. (A) Alignment of the WILYD domains in SUVR2 and its close homologs SUVR1 and SUVR4. The two conserved residues that are subjected to point mutation in this study are labeled with “*”. (B) Alignment of the Pre-SET domains and the SET domains in SUVR1, SUVR2, SUVR4, and their homologs including human G9a, yeast Clr4, and Neurospora Dim-5. The critical catalytic residues are labeled with “*”.
