# Supplementary material for: SUVR2 is involved in transcriptional gene silencing by associating with SNF2-related chromatin-remodeling proteins in Arabidopsis
Source: Cell Res. 2014 Nov 25;24(12):1445–65. doi: 10.1038/cr.2014.156 (PMC4260354; doi:10.1038/cr.2014.156)
Supplement: Supplementary information, Figure S7 — Point mutations in the WIYLD domain and the SET domain of SUVR2 have no effect on transcriptional gene silencing. [file cr2014156x7.pdf]

Fig S7

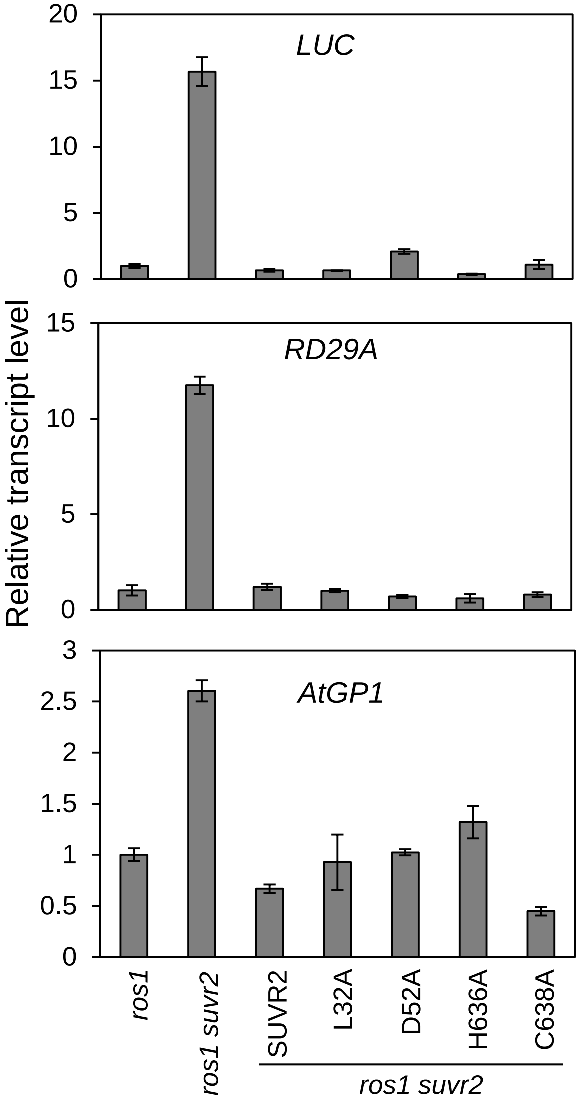


**Supplemental Figure S7. Point mutations in the WIYLD domain and the SET domain of SUVR2 have no effect on transcriptional gene silencing.** The wild-type and mutated *SUVR2* sequences were transformed into *ros1suvr2* to test whether the mutations in the WIYLD and SET domains affect transcriptional gene silencing. The transcript levels of the transgenic *RD29A-LUC* transgene and its corresponding endogenous *RD29A* gene as well as the endogenous retrotransposon *AtGP1* were determined by quantitative RT-PCR.
