# Supplementary material for: SUVR2 is involved in transcriptional gene silencing by associating with SNF2-related chromatin-remodeling proteins in Arabidopsis
Source: Cell Res. 2014 Nov 25;24(12):1445–65. doi: 10.1038/cr.2014.156 (PMC4260354; doi:10.1038/cr.2014.156)
Supplement: Supplementary information, Figure S8 — The interaction of SUVR2 with SUVR2 was not detected by in vitro pull down assays. [file cr2014156x8.pdf]

Fig S8

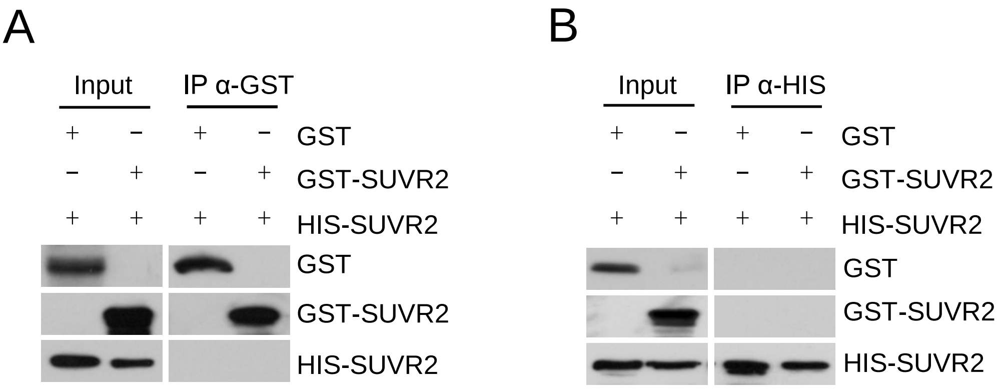

Supplemental Figure S8. The interaction of SUVR2 with SUVR2 was not detected by *in vitro* pull down assays. GST-SUVR2 and HIS-SUVR2 were bacterially expressed and subjected to pull down assays. (A) GST pull down. (B) His pull down. The GST protein was expressed and used as a control.
