# Supplementary material for: SUVR2 is involved in transcriptional gene silencing by associating with SNF2-related chromatin-remodeling proteins in Arabidopsis
Source: Cell Res. 2014 Nov 25;24(12):1445–65. doi: 10.1038/cr.2014.156 (PMC4260354; doi:10.1038/cr.2014.156)
Supplement: Supplementary information, Figure S9 — The full-length SUVR2 and truncated SUVR2 versions have no self-activation activity. [file cr2014156x9.pdf]

AD	BD
Vec	SUVR2
Vec	SUVR2-a
Vec	SUVR2-b
Vec	SUVR2-c
Vec	Vec
Vec	SUVR2-d
Vec	SUVR2-e

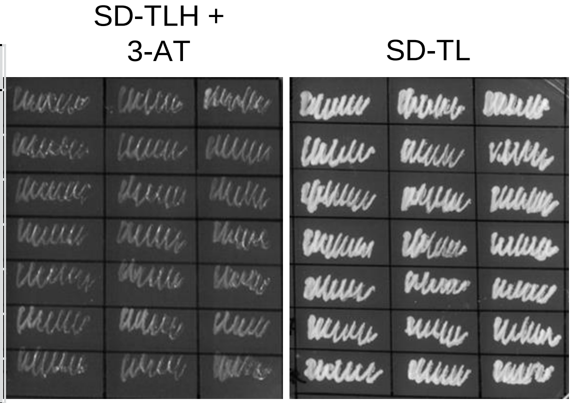

Supplemental Figure S9. The full-length SUVR2 and truncated SUVR2 versions have no self-activation activity. The full-length and truncated SUVR2 versions were separately constructed in the pGBKT7 plasmid. Each of these constructs and the empty pGADT7 plasmid were co-transformed into yeast. The transformed yeast strains were grown on SD-TLH (the synthetic dropout medium minus Trp, Leu, and His) supplemented with 20 mM 3-AT and SD-TL plates for yeast two-hybrid assay.
