# Supplementary material for: SUVR2 is involved in transcriptional gene silencing by associating with SNF2-related chromatin-remodeling proteins in Arabidopsis
Source: Cell Res. 2014 Nov 25;24(12):1445–65. doi: 10.1038/cr.2014.156 (PMC4260354; doi:10.1038/cr.2014.156)
Supplement: Supplementary information, Figure S10 — The function of SUVR1, SUVR2, and the chromatin-remodeling proteins in the silencing of the RdDM target loci ETR7, ETR9, ETR12, and ETR14. [file cr2014156x10.pdf]

Fig S10

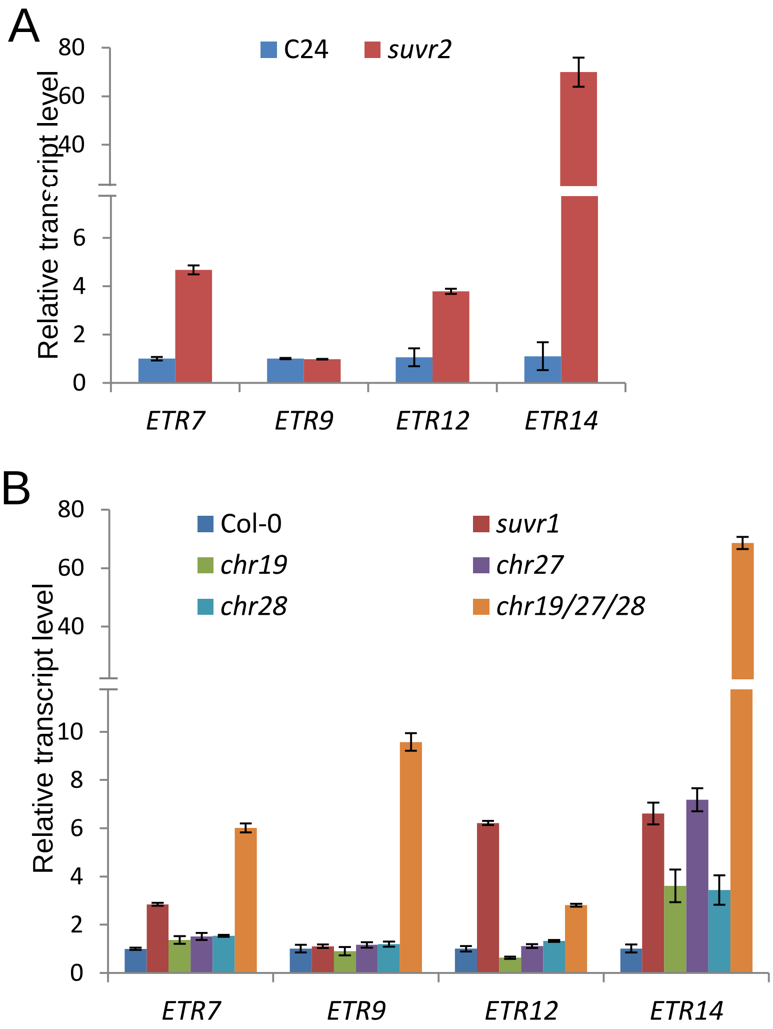

Supplemental Figure S10. The function of SUVR1, SUVR2, and the chromatin-remodeling proteins in the silencing of the RdDM target loci *ETR7*, *ETR9*, *ETR12*, and *ETR14*. (A) The transcript levels of the RdDM target loci in the-wild C24 and *suvr2*. (B) The transcript levels of the RdDM target loci in the wild-type Col-0, *suvr1*, *chr19*, *chr27*, *chr28*, and *chr19/27/28*. The actin gene *ACT2* was amplified as an internal control.
