# Supplementary material for: SUVR2 is involved in transcriptional gene silencing by associating with SNF2-related chromatin-remodeling proteins in Arabidopsis
Source: Cell Res. 2014 Nov 25;24(12):1445–65. doi: 10.1038/cr.2014.156 (PMC4260354; doi:10.1038/cr.2014.156)
Supplement: Supplementary information, Figure S11 — SUVR1 and SUVR2 have a redundant function in the silencing of the RD29A-LUC transgene. [file cr2014156x11.pdf]

Fig S11

C24 *+ LUC*	*ros1suvr1* *+ LUC*	*ros1/* *suvr1suvr2* *+ LUC*
ros1 *+ LUC*	*ros1suvr2* *+ LUC*	*ros1/* *suvr1suvr2* *+ LUC*

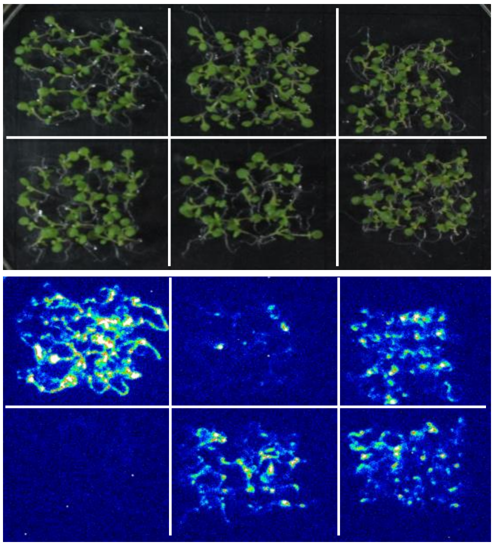

Supplemental Figure S11. SUVR1 and SUVR2 have a redundant function in the silencing of the *RD29A-LUC* transgene. The *suvr1* mutant was crossed with the *ros1* mutant and the *ros1suvr2* mutant, thereby generating the *ros1suvr1* mutant and the *ros1suvr1suvr2* mutant, respectively. The mutants and the wild-type control plants contain the *RD29A-LUC* transgene. The expression of the transgene was indicated by luminescence imaging of seedlings.
