# Supplementary material for: SUVR2 is involved in transcriptional gene silencing by associating with SNF2-related chromatin-remodeling proteins in Arabidopsis
Source: Cell Res. 2014 Nov 25;24(12):1445–65. doi: 10.1038/cr.2014.156 (PMC4260354; doi:10.1038/cr.2014.156)
Supplement: Supplementary information, Figure S12 — The effect of suvr2 and chr19/27/28 on DNA methylation. [file cr2014156x12.pdf]

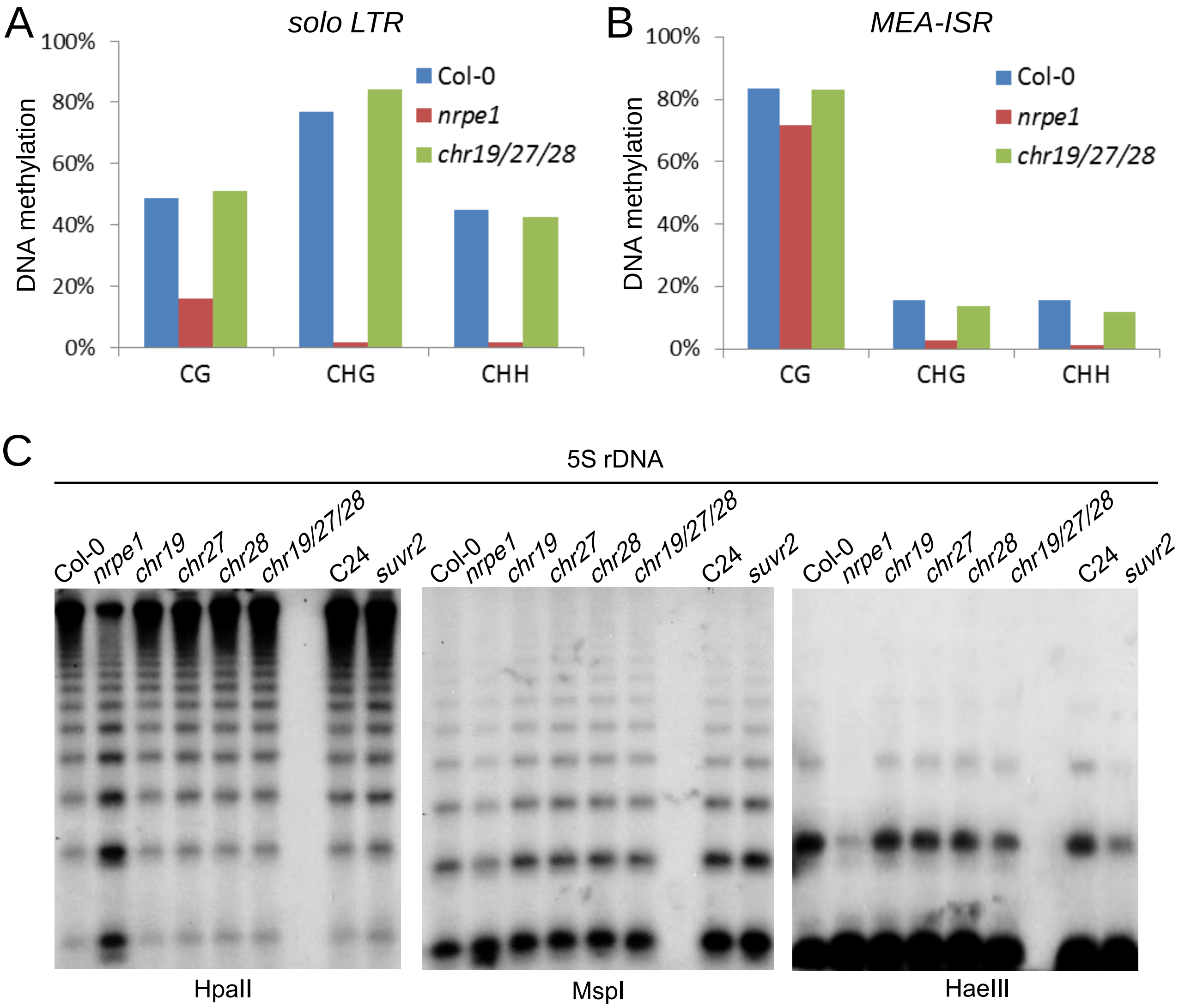

Supplemental Figure S12. The effect of *suvr2* and *chr19/27/28* on DNA methylation. (A, B) DNA methylation was determined by bisulfite sequencing at *solo LTR* (A) and *MEA-ISR* (B) sites in wild-type, *nrpe1*, and *chr19/27/28* plants. Cytosine methylation at CG, CHG, CHH contexts is separately indicated. H represents A, T, and C. (C) *5S rDNA* methylation at CHH sites is weakly reduced by *suvr2* and *chr19/27/28*. Genomic DNA was separately cleaved by the DNA methylation-sensitive restriction enzymes HpaII, MspI, and HaeIII, followed by Southern blotting. HpaII and MspI recognize CCGG but cannot cleave when the external C is methylated. HpaII but not MspI cannot cleave when the internal C is methylated. HaeIII cleaves GGCC but is blocked by the external C methylation. Thus, HpaII, MspI, and HaeIII detect DNA methylation at CG and CHG sites, CHG sites, and CHH sites, respectively.
